# Traffic-light front-of-pack environmental labelling across food categories triggers more environmentally friendly food choices: a randomised controlled trial in virtual reality supermarket

**DOI:** 10.1186/s12966-023-01410-8

**Published:** 2023-01-26

**Authors:** Laura Arrazat, Stéphanie Chambaron, Gaëlle Arvisenet, Isabelle Goisbault, Jean-Christophe Charrier, Sophie Nicklaus, Lucile Marty

**Affiliations:** 1 Centre Des Sciences Du Goût Et de L’Alimentation, CNRS, INRAE, Institut Agro, Université Bourgogne Franche-Comté, 17 Rue Sully, 21065 Dijon Cedex, France; 2 Strategir – R&D and Image and Technology Department, 5 Rue Foy, 33000 Bordeaux, France

**Keywords:** Environmental labelling, Food choice, Supermarket, Virtual reality

## Abstract

**Background:**

Food systems highly contribute to anthropogenic greenhouse gas emissions and shifting towards more environmentally friendly diets is urgently needed. Enabling consumers to compare the environmental impact of food products at point-of-purchase with front-of-pack labelling could be a promising strategy to trigger more environmentally friendly food choices. This strategy remained to be tested.

**Methods:**

The effect of a new traffic-light front-of-pack environmental label on food choices was tested in a 2-arm randomised controlled trial in a virtual reality supermarket. Participants (*n* = 132) chose food products to compose two main meals for an everyday meal scenario and for an environmentally friendly meal scenario with or without the label. The environmental label (ranging from A: green/lowest impact, to E: red/highest impact) was based on the Environmental Footprint (EF) single score calculation across food categories. The effect of the label on the environmental impact of food choices in each scenario was tested using linear mixed models.

**Results:**

In the everyday meal scenario, the environmental impact of meals was lower in the label condition than in the no label condition (-0.17 ± 0.07 mPt/kg, *p* = 0.012). This reduction was observed at no nutritional, financial nor hedonic cost. The effectiveness of the label can be attributed to a change in the food categories chosen: less meat-based and more vegetarian meals were chosen with the label. In the environmentally friendly meal scenario, we demonstrated that the label provided new information to the participants as they were able to further reduce the environmental impact of their food choices with the label (-0.19 ± 0.07 mPt/kg, *p* = 0.005).

**Conclusions:**

Implementing a front-of-pack environmental label on food products in real supermarkets could increase awareness of the environmental impact of food and contribute to drive more environmentally friendly food choices.

**Trial registration:**

The study protocol was pre-registered prior to data collection at Clinicaltrials.gov (NCT04909372).

**Supplementary Information:**

The online version contains supplementary material available at 10.1186/s12966-023-01410-8.

## Background

Food systems play a major role in anthropogenic greenhouse gas emissions (GHGE) [1], threatening the achievement of limiting the increase in global temperatures to 2 °C as targeted by the Paris Agreement [2]. Beyond technological solutions to improve energy efficiency in food production practices and waste reduction, diet change has been advocated as a necessity to remain within a safe operating space for food systems encompassing human health and environmental sustainability [3, 4]. In developed countries, meat products are the highest contributors to diets’ GHGE [5, 6] and have been shown to have multiple negative impacts on both human health and the environment [7]. Yet, a shift towards sustainable diets including a diversity of plant-based foods and low amounts of animal source foods may not be easily achieved [8], meat consumption being central in meal composition in western food cultures and associated with pleasure, as well as social, personal and cultural values [9, 10].

In this context, highlighting the need for a global public awareness of the environmental impacts of food products, the French government has set up an interdisciplinary committee to assess the feasibility and efficiency of environmental labelling on food products [11]. The present study was carried out as part of the national experimentation phase (2020–2021) that aimed to inform the implementation of an environmental label in the food sector in France as announced by the French government in February 2020 [12]. Providing information at the point of choice about the environmental impact of food products could overcome a gap in knowledge as consumers’ underestimation of the environmental impact of food products, especially of meat products, is common [13, 14]. Easily understandable environmental labels on food products have been advocated as a simple public policy instrument to unfold, in order to alter food choice behaviours and increase their sustainability [15, 16].

A recent systematic review by Potter and colleagues (2021) provided promising evidence regarding the ability of carbon footprint labels or other ecolabels (e.g., organic, sustainable agricultural practices) to promote more environmentally friendly food choices [17]. However, few studies were randomised controlled trials where the aim of the study was blinded to participants, i.e. the gold standard methodology to test the effect of an intervention. We identified only three studies that tested the effect of labels depicting the environmental impact of food products in a between-subject design with a control group [14, 18, 19]; and the designs included a very limited range of products (canned soups, pasta dishes or beefsteak, chicken breast and veggie burger) which did not reflect the substantial shifts required to adopt sustainable healthy dietary patterns. To our knowledge, no study has tested the effect of a front-of-pack environmental label across various food categories (i.e. red meat, poultry, fish, vegetables, starchy foods, pulses, etc.) which is the most likely strategy to be implemented at country or cross-country level based on nutritional labelling precedents [20]. Yet, it might be difficult for consumers to shift from one category to another even aided by labels; for instance, meat is central in the composition of meals and choosing more environmentally-friendly options resulting in a meal without meat requires a deep change in consumers’ culinary scripts [10].

Another limitation of previous studies is their designs that may have increased participants’ attention to environment labels compared to real-life [17]. A way of addressing this visual bias is to use a virtual reality (VR) environment that mimics real-life prominence of environmental labels [21]. With VR technology, participants are immersed in a 3D virtual environment that interacts with them in real-time while providing great control of the experimental design such as an identical food choice environment for each participant [22]. Virtual supermarkets have been validated as an appropriate tool to measure food choices and purchases [23–25].

The present study was a randomised controlled trial with two experimental arms (with vs. without environmental labelling) conducted in a virtual reality supermarket that included a large and diverse range of food products that could be part of a main meal. We aimed to develop a new traffic-light front-of-pack environmental label with a design based on state-of-the-art evidence [26–28] and that differentiated food products across food categories, to support between-categories substitutions. Participants performed food choice tasks for an everyday meal and for an environmentally friendly meal. The latter had the objective to highlight whether the label provided new information about the environmental impact of food products compared to the level of existing knowledge.

## Methods

### Data collection

The study protocol was pre-registered prior to data collection at Clinicaltrials.gov (NCT04909372). Participants were recruited using the PanelSens database declared to the relevant French authority (*Commission Nationale Informatique et Libertés*; CNIL; n°1,148,039). To be eligible for this study, participants were to be aged between 18 and 65 years, to be responsible for a substantial proportion of household grocery shopping, to be fluent in French, to have no dietary restrictions, to have no uncorrected eye problems and not to be aware of symptoms of dizziness when wearing a virtual reality headset. Recruitment was stratified by gender (50% male, 50% female) and age (33% 18–35 years, 33% 36–50 and 33% 51–65). A 10€ voucher was given to the participants who completed the study. The study was approved by the CEEI-IRB ethical committee (N°21–780, Institutional Review Board INSERM) and was led in accordance with the Declaration of Helsinki. Data were collected in a laboratory setting in France between May and June 2021.

### Study design

**Fig. 1:**
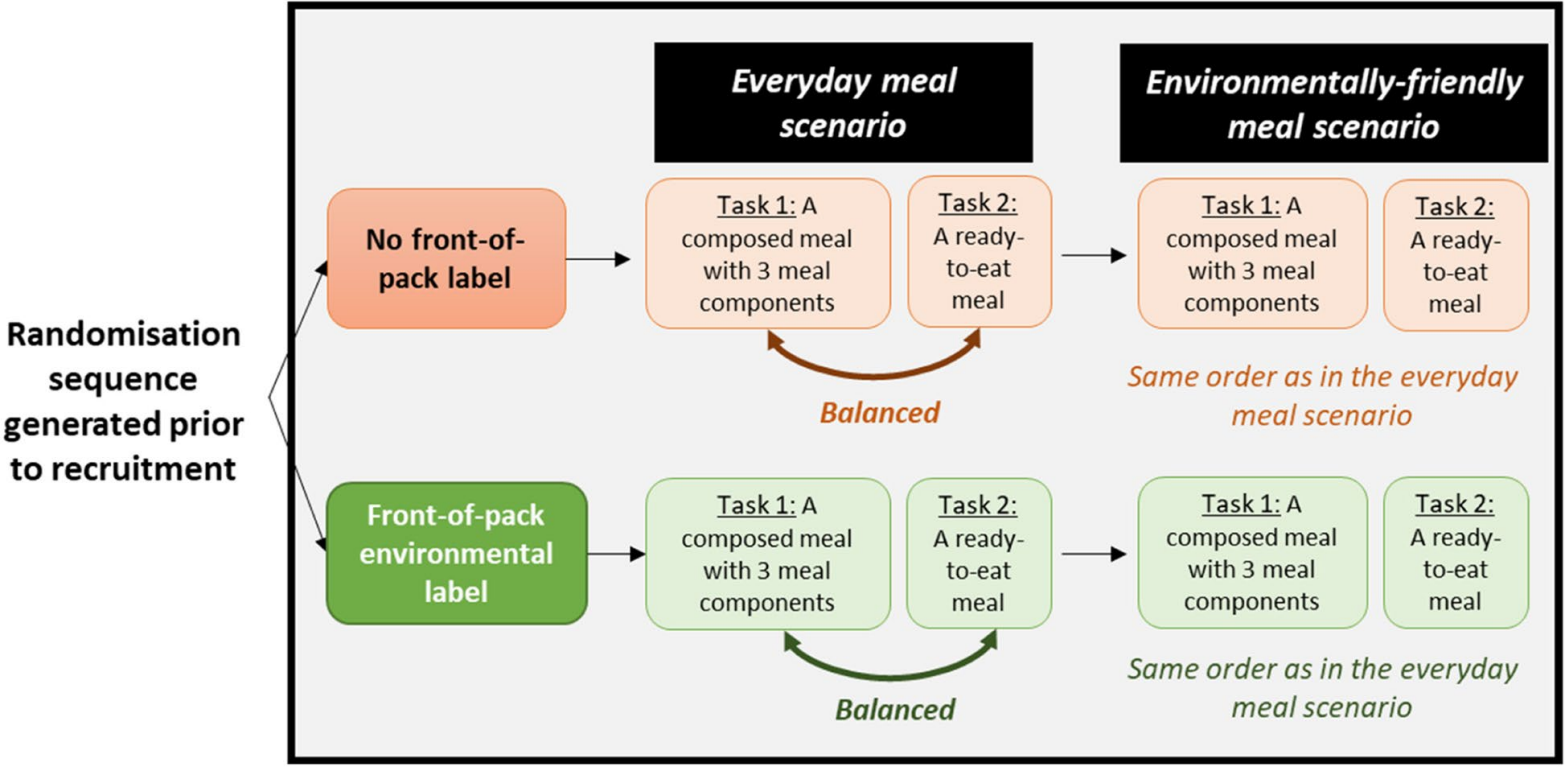
Experimental design for the virtual reality tasks performed by the participants

This study was a randomised controlled trial in a virtual reality supermarket with two experimental arms: (1) no front-of-pack environmental labelling (no label condition) and (2) front-of-pack environmental labelling (environmental label condition). A 1:1 2-block randomisation sequence (for male and female) was generated before recruitment using the Random Allocation Software [29] and research assistants were blinded to the labelling condition in which each participant was allocated. Regardless of the labelling condition, each participant in the virtual supermarket performed two tasks: 1/ choice of three meal components among 66 products (e.g., lentils, carrots, ham, egg) for a composed meal, 2/ choice of one ready-to-eat meal among 30 products (e.g., beef lasagne, risotto). These two tasks were performed twice: for an everyday meal (everyday meal scenario) and for a meal that was “good for the planet” (environmentally friendly meal scenario). The order of the two tasks was balanced within each scenario. The experimental design is presented in Fig. 1.

### Procedure

Individual experimental sessions lasted on average 35 min (min: 25 min, max: 45 min). After participants gave their written informed consent to take part in the experiment, they sat on a chair and were equipped with a virtual reality (VR) headset by a research assistant. Participants were given instructions regarding the VR headset and hand controller (with a joystick and selection buttons). Pictures of the virtual environment and of the VR headset and hand controller are presented in supplementary file (Figure S1). Participants first tested the functionalities of the VR headset in a dummy supermarket shelf with fake hair-care products. When ready, participants carried out the four food choice tasks in a row. Participants allocated to the environmental label condition were presented with the environmental label before the food choice tasks with a pop-up message (see supplementary file, Note S1). After completing the four tasks, the participants were asked to evaluate their familiarity and liking for the eight products chosen. Then, they removed the VR headset and answered three questionnaires on a computer in the same room. At the end of the session, the research assistant revealed the aim of the study and gave the participant a 10 € voucher.

### Virtual supermarket environment

#### Virtual reality functionalities

The virtual supermarket environment was inspired by recent work by Melendrez-Ruiz and colleagues (2021) [30] and was designed by the firm Strategir (https://www.strategir.com/fr/) using Unity software under the VR headset Oculus Quest 2. Participants saw real size packaged food products on the supermarket shelves. With a hand controller, the participants were able to navigate the shelves, seize and examine products. When a product was seized, it could either be selected and put in the shopping basket or be placed back on the shelf.

#### Food products

Based on the results of a recent study in a virtual reality supermarket using similar food choice tasks [30], four food categories were included: (1) meat, egg, fish and plant-based substitutes, (2) pulses, (3) vegetables and (4) starchy foods. The food products included in each category were selected from real food products available in French supermarkets at the time of the study. As participants were asked to select food products for the main meal, only savoury food products that are typically part of a French main meal were included. Food products were chosen with contrasted level of environmental impact. Two shelves were created with 96 food products in total: 66 meal components (Task 1) and 30 ready-to-eat meals (Task 2). The 66 meal components were starchy foods (*N* = 12), vegetables (*N* = 12), pulses (*N* = 12), beef and lamb (*N* = 6), poultry and pork (N = 6), fish (*N* = 6), dairy products and egg (*N* = 6) and plant-based products (*N* = 6). The meal components were either canned, dry or fresh and needed to be combined to compose the main meal (e.g. fish filet, humus, carrots, salad, canned beans or ham). The 30 ready-to-eat meals were beef- or lamb-based (*N* = 6), poultry- or pork-based (*N* = 6), fish-based (*N* = 6), dairy product- or egg-based (*N* = 6) and plant-based (*N* = 6). Ready-to-eat meals were either fresh or frozen and constituted the main meal as a whole. The products arrangement was based on field observations in French supermarkets (see supplementary file, Figure S2). To control for availability effect, the same space was allocated to each product within a shelf. The 96 food products selected were bought from real supermarkets and photographed in high definition. Expiry dates, prices and sales labels were removed.

#### Recorded data

Food choices were recorded for each task within the VR headset. Each participant had to rate the eight food products selected for their level of familiarity (frequency of consumption of a similar product, ranging from 1 = “never” to 5 = “very often”) and liking (continuous sale, ranging from 1 = “I do not like [the food product] at all” to 10 = “I like [the food product] very much”).

### Environmental label

#### Calculation of the environmental score

The environmental impact, measured by the Environmental Footprint single score (EF single score), of the 96 foods items included in the virtual supermarket was retrieved from the open-access Agribalyse database [31]. The EF single score has been recommended by the European Commission when studying the environmental impact of food products [32]. It aggregates 16 indicators derived from life cycle analysis (see supplementary file for details on the EF single score calculation, Note S2). We also considered three individual indicators retrieved from the Agribalyse database: greenhouse gas emissions (kg CO2 eq/kg of food product), ozone depletion (kg CFC-11 eq/kg of food product) and particulate matter (disease incidence/kg of food product) for sensitivity analyses. These three individual indicators had the highest robustness in the Agribalyse database [31]. A five-level environmental impact score (ranging from A to E) was calculated based on the EF single score of the products. Levels cut-offs were calculated separately for the 66 meal components and the 30 ready-to-eat meals. They were defined as the quintile values from a larger selection of similar foods from the Agribalyse database. Details regarding the five-level environmental impact score calculation are presented in supplementary file (Note S3). The food products were distributed in the five environmental impact scores as follows for meal components: A (*N* = 27), B (*N* = 15), C (*N* = 7), D (*N* = 8) and E (*N* = 9) and for ready-to-eat meals: A (*N* = 4), B (*N* = 7), C (*N* = 7), D (*N* = 7) and E (*N* = 5).

#### Design of the environmental label

**Fig. 2:**
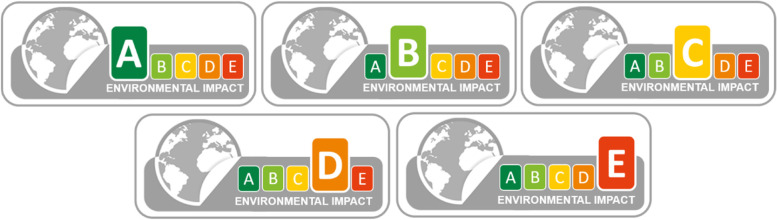
Design of the traffic-light front-of-pack environmental labels

As there was neither an existing environmental label nor a consensus on an environmental label design for food products in France at the time of the present study (May 2021), we designed a new label. The ideal carbon label proposed by Carrero et al., 2021 is a traffic-light label. It has been shown that green is often seen as a validation and a positive colour whereas red is associated to negative aspects and danger [27]. As traffic-light labels are the most effective type of labels to increase understanding and promote behaviour change [28], the French blue label depicting the environmental impact of products or services (other than food) was adapted to obtain a traffic light label (see supplementary file, Note S4). The final environmental label depicted five levels of environmental impact, from A (the lowest) to E (the highest), that were based on the Environmental Footprint (EF) single score calculated for each product (Fig. 2).

### Additional questionnaires

Participants were asked to complete questionnaires on Qualtrics survey platform (www.qualtrics.com) at the end of the experimental session. Participants were asked to fill in an investigation questionnaire that aimed to identify aim-guessers and to record participants’ feedback about their experience in the virtual reality supermarket. We asked questions regarding how easy it was to use the different functionalities of the VR headset, participant’s sensation of being in a real supermarket and if participants thought the selection of products was realistic (4-point Likert scale from “strongly disagree” to “strongly agree”). Participants were also asked whether they paid attention, understood and used the environmental labels while selecting food products. Finally, they completed a socio-demographic questionnaire.

### Outcomes

#### Primary outcome

The primary outcome was the environmental impact (EF single score in mPt per kg of product) of the food products selected in each task. The environmental impact of the composed meal for Task 1 was calculated as the mean of the EF single scores per kg of the three selected meal components in each scenario. The environmental impact of the ready-to-eat meal for Task 2 of each scenario was defined as the EF single score per kg of the selected product. Other indicators were calculated in the same manner: greenhouse gas emissions (GHGE), ozone depletion and particulate matter.

#### Secondary outcomes

The nutritional quality of the food products selected was assessed by the FSA score developed by the British Food Standards Agency [33]. A food product’s FSA score is calculated by allocating positive points for unfavourable nutrients (energy, saturated fatty acids, total sugar and sodium) and negative points for favourable nutrients or food groups (protein, fibre, fruits, vegetables, legumes and nuts). The final FSA score ranges from -15 points (best nutritional quality) to + 40 points (worst nutritional quality). The nutritional quality of the product selection for Task 1 was calculated as the mean of the FSA scores for 100 g of the three meal components selected in each scenario. The nutritional quality of the ready-to-eat meal selected for Task 2 of each scenario was defined as the FSA score for 100 g of the selected product. Actual prices displayed in supermarkets at the time of the study and energy densities from the food products packaging were used for the calculation of the energy cost. Energy cost of the composed meal was calculated as the mean of the price per kcal of the three meal components selected in each scenario. The energy cost of the ready-to-eat meal selected for task 2 was defined as the price per kcal of the selected product. Liking and familiarity scores of the composed meal were calculated as the average of the liking and familiarity scores for the three meal components, respectively.

#### Meal types

Meals types were defined based on the source of protein that was included in a meal: meat-meals (further distinction between beef/lamb meals and poultry/pork meals), fish meals and vegetarian meals (further distinction between lacto-ovo meals or plant-based meals). In total, there were three main meal types (meat-based, fish-based and vegetarian meals) and five sub-meal types (beef or lamb-based, poultry or pork-based, fish-based, lacto-ovo and plant-based meals). We ranked the sources of protein on the basis of their environmental impact: beef or lamb > poultry or pork > fish > dairy or egg > plant-sourced protein [7]. When a meal was composed of more than one protein source, the meal was classified within the most emitting one (i.e. beef lasagne contained dairy and beef and were classified as a beef or lamb-based meal).

### Statistical analyses

We followed an analysis plan pre-registered on Open Science Framework (https://osf.io/du58k/). Any minor changes from the pre-registered analytic plan are described in supplementary file (Table S1). All the statistical analyses were performed using SAS version 9.4. The level of significance was set at *p* < 0.05 for all the analyses.

EF single scores were standardised within the 66 meal components and the 30 ready-to-eat meals shelves because the scores of food products composing each shelf had different distributions (average environmental impact for the meal components shelf: 0.61 ± 0.86, ready-to-eat meals shelf: 0.54 ± 0.39). The standardisation allowed the comparison of the two tasks in the same statistical model. To examine the effect of the environmental label on the environmental impact of food choices, the primary analysis consisted in a mixed model that tested the effects of labelling (categorical variable: environmental label or no label), food choice task (categorical variable: choice of a composed meal or a ready-to-eat meal), labelling*food choice task interaction on standardised EF single scores (everyday meal scenario only), with random effect of participants to account for correlation between repeated measures. Parameters estimates were calculated after removing non-significant interaction.

In sensitivity analyses, we tested whether the results from the primary mixed model differed: 1/ after excluding aim-guessers, 2/ after excluding outliers on the primary outcome, 3/ after adjusting for age, gender, highest education level and BMI and 4/ after replacing standardised EF single scores by standardised GHGEs, ozone depletion and particulate matter scores.

To examine existing knowledge and additional information provided by the environmental label, the main effect of food choice scenario (categorical variable: everyday meal scenario or environmentally friendly meal scenario), as well as labelling*scenario and scenario*food choice task interactions, were added to the primary mixed model presented above. Pairwise post-hoc comparisons were conducted to compare the environmental impact of food choice between the everyday meal and environmentally friendly meal scenario in participants with no label (i.e., highlighting existing knowledge) and to compare the environmental impact of food choice between no label and environmental label condition in the environmentally friendly meal scenario (i.e., highlighting additional information provided by the label). Parameters estimates were calculated after removing non-significant interactions.

As secondary analyses we examined the effect of the environmental label on secondary outcomes with mixed models testing the effect of labelling, food choice task and labelling*food choice task interaction on nutritional quality, price per kcal, liking and familiarity (everyday meal scenario only), with random effect of participants to account for correlation between repeated measures.

As exploratory analyses, Chi-squared tests were performed to test whether labelling condition was associated to meal types (beef or lamb-based meals, poultry or pork-based meals, fish-based meals, dairy or egg-based meals and plant-based meals) in the everyday meal scenario for Task 1 (composed meal) and Task 2 (ready-to-eat meal) separately.

### Sample size

We powered the primary analyses in order to detect a d = 0.50 effect size of labelling condition on EF single scores based on the results of a previous randomised controlled trial that tested the effect of an environmental label on canned soup choices [14]. A sample size of 122 participants was required for 80% power at α = 0.05 (SAS 9.4). We aimed to recruit a sample of 130 participants to account for potential data loss due to technical problems and drop-outs.

## Results

### Participants

**Fig. 3:**
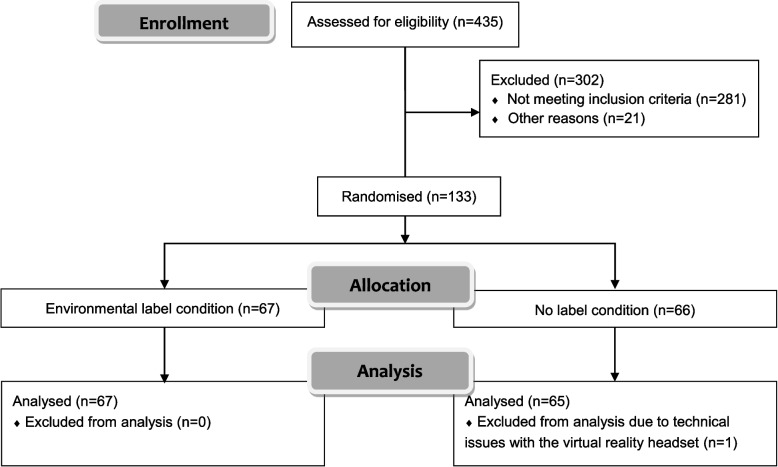
CONSORT flow diagram

A total of 435 participants were assessed for eligibility and data from 132 who completed the study were analysed (Fig. 3). Participants’ characteristics are reported in supplementary file (Table S2). No significant difference was found between the participants randomised in each experimental condition.

### Effect of labelling on environmental impact of everyday meals

**Fig. 4:**
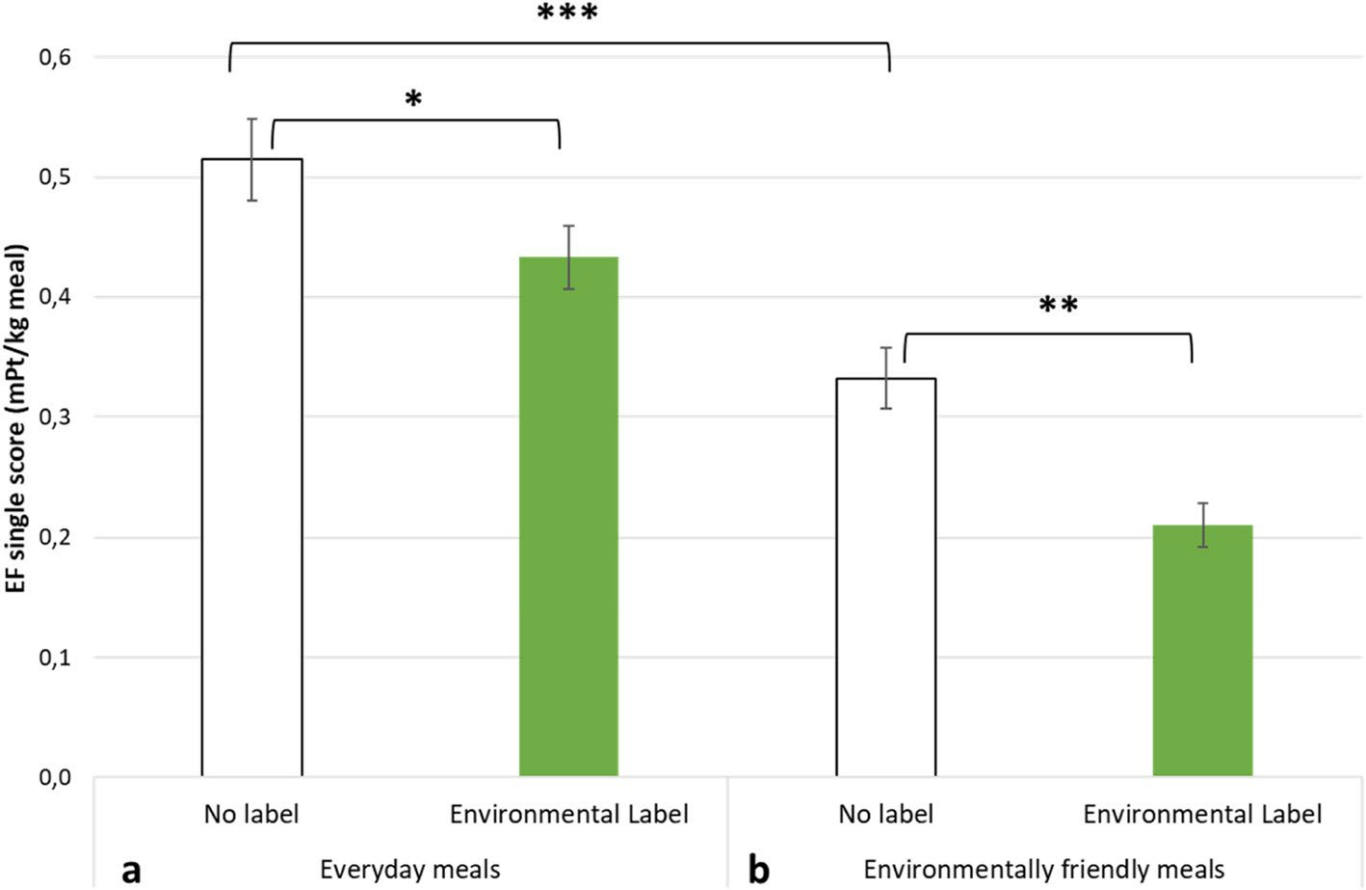
Mean (± SEM) of EF single scores for food products selection in the two tasks combined (choice of a composed meal and a ready-to-eat meal), in the two food choice scenarios (everyday meal or environmentally friendly meal), with or without environmental label. * *p* ≤ 0.05, ** *p* ≤ 0.01 and *** *p* < 0.001 for least-square means post-hoc tests on standardised EF single scores

The participants were first asked to choose food products for an everyday main meal, in two distinct tasks in the virtual reality supermarket. During the first task, they selected three meal components for a composed meal and during the second task, they selected a ready-to-eat meal. Participants randomised in the environmental label condition (*n* = 67) saw exactly the same products in the virtual supermarket as participants randomised in the no label condition (*n* = 65), except that an environmental label was added on the front of the packaging of each product. In a linear mixed model, we found significant effects of the labelling condition (-0.17, t(131) = -2.22, *p* = 0.028) and of the food choice task (-0.37, t(131) = -5.34, *p* < 0.001) on standardised EF single scores of the food products that were selected, but no significant interaction between the environmental label and the food choice task (see supplementary file, Table S3). This non-significant interaction indicates that the environmental label was equally effective in reducing the environmental impact of food selection in the meal components shelf as in the ready-to-eat meals shelf. For this reason, data from the two tasks were combined in Fig. 4. Participants chose food products for everyday meals of lower environmental impact when exposed to the environmental label compared to the no label condition (Fig. 4a). The same pattern of results was observed in sensitivity analyses when removing aim-guessers or outliers from the analyses, adjusting for socio-demographic characteristics and replicating the model on other environmental impact indicators: GHGE, ozone depletion and particulate matter (see supplementary file, Table S4).

**Fig. 5:**
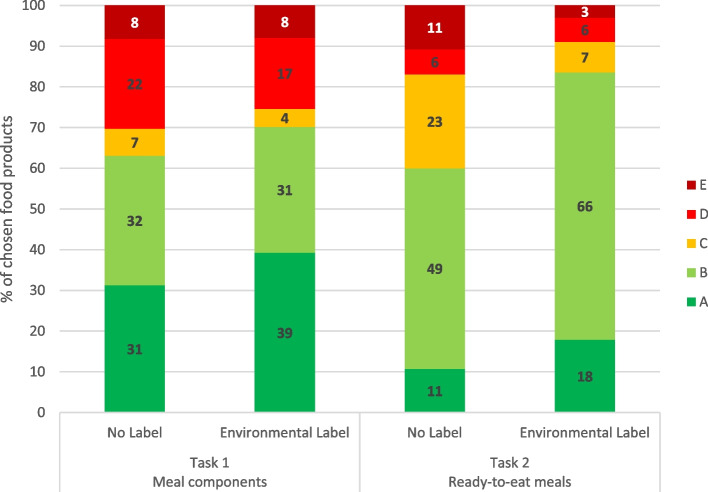
Food choices based on the five environmental impact scores (from A to E) for meal components and ready-to-eat meals, in the everyday meal scenario, with or without environmental label

For descriptive purpose, Fig. 5 shows the percentage of food products chosen for each task based on their environmental impact score as displayed on the front-of-pack labels.

### Effect of labelling on the environmental impact of environmentally friendly meals

After having selected food products for an everyday meal, all the participants were asked to choose food products for an environmentally friendly meal. In a linear mixed model, we found a main effect of the labelling condition (-0.18, t(394) = -3.44, *p* < 0.001), food choice task (-0.34, t(394) = -8.11 *p* < 0.001) and food choice scenario (-0.31, t(394) = -7.40, *p* < 0.001) on standardised EF single scores but no significant interactions between these variables (see supplementary file, Table S5). The significant main effect of the food choice scenario indicates that participants chose food products with a lower environmental impact when asked to compose an environmentally friendly meal compared to when asked to compose an everyday meal, independently of the labelling condition or of the food choice task. For participants in the no label condition, we compared EF single scores of food products selection between the everyday meal scenario and the environmentally friendly meal scenario and found that consumers were able to reduce the environmental impact of their meals without the presence of any label (Fig. 4a and 4b). This result highlights existing knowledge about the environmental impact of food products that were retrieved by the participants to select environmentally friendly meals. In addition, we compared EF single scores of food products selection between labelling conditions in the environmentally friendly meal scenario and found that food choices in the environmental label condition were of significantly lower environmental impact than in the no label condition (Fig. 4b). This result highlights that the environmental label increased the ability of the participants to identify more environmentally friendly food products by providing new information regarding the environmental impact of the food products in addition to existing knowledge.

### Meal types selection

We showed that participants in the environmental labelling condition selected everyday meals of lower environmental impact than participants in the no label condition. We further investigated whether the cross-category nature of the environmental label led to a lower environmental impact of food choices through between-categories substitutions. In exploratory analyses (not pre-registered), we thus compared everyday meal choices classified into three meal types: meat-based meals (with a further distinction between beef/lamb and poultry/pork), fish-based meals and vegetarian meals (with a further distinction between lacto-ovo vegetarian meals and plant-based meals) categories.

**Fig. 6:**
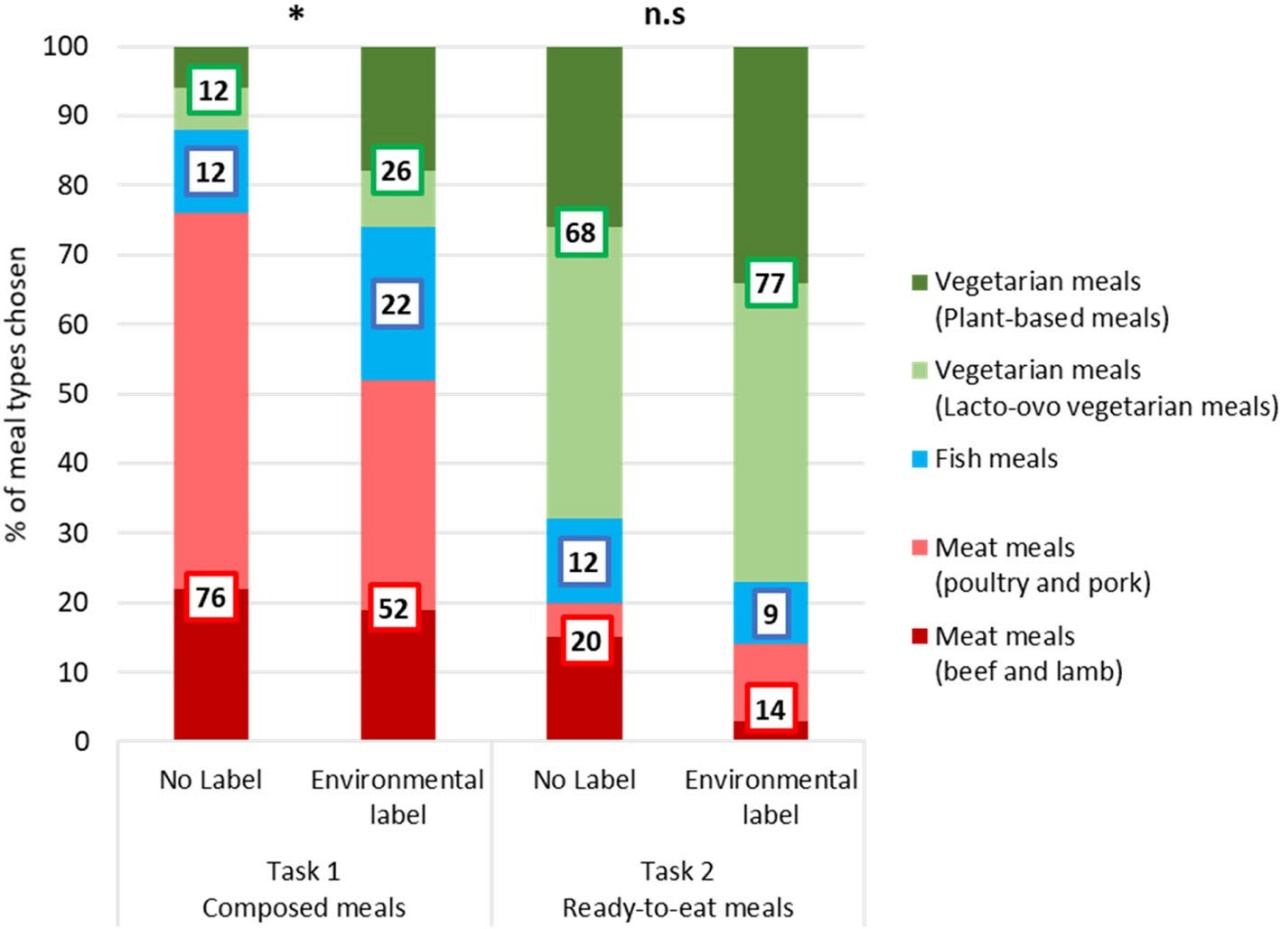
Meal types choices for the composed meals and ready-to-eat meals in the everyday meal scenario, with or without environmental label. (Chi-2 test * *p* < 0.05)

Meal types chosen for the composed meals were significantly influenced by the label (Chi-2 = 7.67, *p* = 0.021) but choices for the ready-to-eat meals were not (Chi-2 = 1.65, *p* = 0.438) (Fig. 6). The environmental label led to between categories substitutions when choosing food products to compose a meal but on the contrary, in the case of ready-to-eat meals, substitutions leading to a lower environmental impact were not between-categories and thus likely happened within-categories.

### Nutritional quality, energy cost, familiarity and liking

In a linear mixed model, there were no significant effects of the environmental label on nutritional quality (-0.12, t(131) = -1.29, *p* = 0.200), energy cost (0.10, t(131) = 0.86, *p* = 0.394), familiarity (-0.05, t(131) = -0.39, *p* = 0.696) and liking score (0.03, t(131) = 0.17, *p* = 0.868) of the everyday meal, as tested by linear mixed models also including fixed effect of food choice task. Further details on the models are presented in supplementary materials (Table S6). These results show that traffic-light front-of-pack environmental labels led to more environmentally friendly food choices without altering their nutritional quality, familiarity and liking nor increasing the cost per kcal. This suggests good acceptability of such food choices by the consumers.

### Perception of the label and virtual reality experience

Participants in the labelling condition were asked whether they noticed and understood the environmental label on the food products when they were in the virtual supermarket. Results showed that 89% declared having seen the environmental label and 96% that the environmental label depicted the environmental impact of the food products (see supplementary materials, Table S7). In addition, the majority of the participants found the virtual supermarket easy to use (98%), attested feeling immersed in a supermarket (99%) and agreed that the products were similar to those found in real supermarkets (96%). Detailed results are described in supplementary materials (Table S8).

## Discussion

For the first time, the effectiveness of traffic-light front-of-pack environmental labelling was tested in a randomised controlled trial conducted in a virtual reality supermarket, including a large and diverse range of food products. We demonstrated that the environmental label significantly reduced the environmental impact of food choices at no nutritional, financial or hedonic cost. In the present study, we developed a cross-category environmental label which has been highlighted as a promising tool to substantially reduce the environmental impact of diets [34]. By ranking all the food products along the same scale, cross-category labels could help consumers building a broad understanding of the environmental impact of food products and identifying what changes in diet would be the most beneficial for the environment, notably a reduction in meat consumption. In line with this idea, we observed that the environmental label enhanced the ability to identify food products with a lower environmental impact highlighting its capacity to provide additional information to consumers. Participants also had some existing knowledge regarding the environmental impact of food products that were not necessarily used to choose an everyday meal but may have been retrieved thanks to the presence of the label at the point of choice. The presence of the label resulted in a shift towards the selection of more vegetarian meals (composed meals) which was in line with the expected effect of cross-category environmental labelling. We thus complemented previous research that showed a convincing effect of environmental labelling on food choices mostly within a given category of food products [17], by highlighting that environmental labelling was also able to trigger changes across food categories which may lead to the necessary reduction in consumption of animal source foods to substantially reduce the environmental impact of food systems [3, 7]. Nonetheless, as previous research work has identified lack of knowledge but also convenience as barriers to a reduction of meat consumption [35, 36], training in culinary skills should accompany the spread of a front-of-pack environmental label across food categories to facilitate the choice of meat alternatives.

**Fig. 7:**
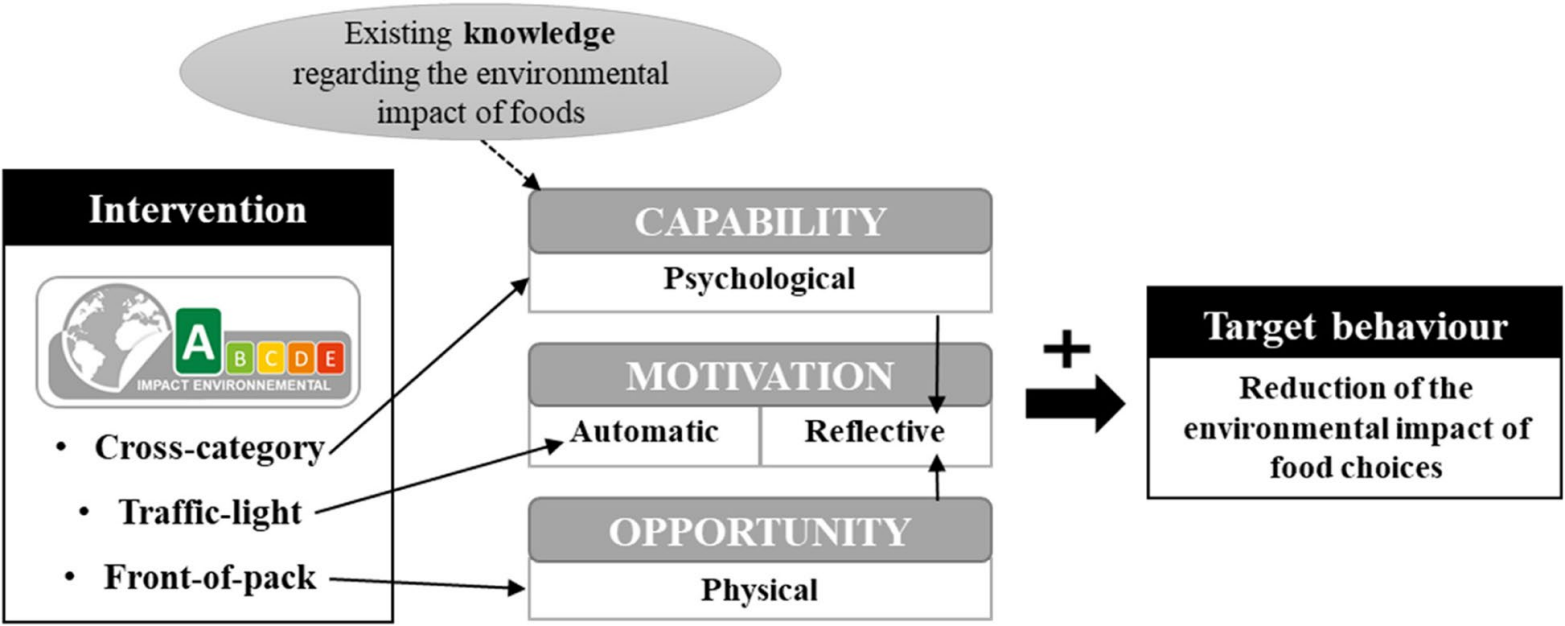
Application of the COM-B framework to a cross-category traffic-light front-of-pack environmental label

As discussed in a recent review on the effect of environmental labels and based on the COM-B (Capacity Opportunity Motivation Behaviour) framework for understanding behaviour [17, 37], mechanisms through which cross-category traffic-light front-of-pack environmental labels could promote a shift toward food choices of lower environmental impact, and in particular less meat-based and more plant-based diets, are threefold. The three characteristics of the environmental label we used (i.e. cross-category, traffic-light and front-of-pack) specifically targeted each behavioural pathway to achieve the target behaviour and are summarized Fig. 7. First, capability could have been enhanced by the cross-category aspect of the label by providing information to compare the environmental impact of food products, in particular meat-based and plant-based products. Second, the opportunity to substitute a high environmental impact (e.g., meat-based) by a low environmental impact food product (e.g., plant-based) could have been increased because all of the products included front-of-pack environmental impact information at point-of-choice. Third, automatic motivation to choose more products with a low environmental impact and less products with a high environmental impact could have been enhanced by the traffic-light colour coding, as green labels are seen as a validation whereas red labels are associated with danger [27]. In line with this theoretical framework, the promising effect of the traffic-light front-of-pack environmental label reported here leans on the fact that the label we developed was present on all the food products (hence noticed), well understood (because it was based on an already existing labelling layout) and resulted in an enhanced ability to spot food products with a low environmental impact.

In the context of a national experimentation phase of environmental labelling in the food sector (2020–2021) coordinated by the French Agency for Ecological Transition (ADEME), the results of the present study contributed to inform the development and future implementation of an environmental labelling system for all food products in France. Numerous stakeholders and experts contributed to this experimentation phase providing guidelines on six aspects 1/ environmental issues that should be considered, 2/ objectives that should be targeted, 3/ data that are needed, 4/ methods for assessing environmental impacts, 5/ environmental scores that should be chosen, 6/ label format that should be proposed [38]. Specifically, the present study highlighted that a cross-category traffic-light front-of-pack environmental label that is unique, well understood and informative was effective in reducing the environmental impact of food choices. In real life, meeting all these criteria raises substantial challenges for policy makers, in line with drawbacks previously described for environmental labelling implementation in other sectors [39, 40]. First, a unique label voted in by the agricultural and food industry is needed to guarantee its widespread and to allow comparisons between different food products and different food categories. Second, the label needs to be trustworthy and well understood, implying the establishment of an independent structure engaging with scientific experts, organising co-creation phases with citizens and broadcasting a communication plan at a national level. Third, there is a need to take into account the potential joint effect of a new environmental label with existing labels on food products, e.g. nutritional or organic labels [41]. To date, no country in the world has voted on a unique environmental label across all food products. Bearing in mind these challenges, the present work adds to a set of promising international scientific evidence highlighting the effectiveness of environmental labelling in lowering the environmental impact of food choices [15, 17] which should support the French government in taking a step further in the process of introducing environmental labelling for all food products.

### Strengths and limitations

This study was a pre-registered randomised controlled trial in a realistic food choice environment and the first to test the effect of an environmental label on food choices in a virtual supermarket. Using questionnaires, we confirmed that the label had been seen, understood and used by a majority of the participants in the labelling condition.

Our study is unique compared to the previous studies that have tested environmental labelling in online supermarkets as consumers may not have the same shopping behaviours in (virtual) stores compared to online. In particular, it has been shown that contrary to online food environments where factual information is more important, sensory attributes are stronger drivers of in-store food choices [42]. A strength of using virtual reality that reproduced real-life environments is that hypotheses can be tested under realistic yet tightly controlled conditions. However, participants were not asked to spend their own money nor to consume what they selected which limits the strength of our results although other research has shown that food choice tasks using virtual environments are valid to adequately evaluate food purchasing behaviours [43, 44].

We could only include a limited number of food items in the virtual supermarket. We decided to only include items that could be part of a main dish because meat-based products are the food items with the worst impact on the environment and are usually consumed as part of main dishes [5, 7]. Consequently, we may have overestimated the effect of the label in our design compared to a shopping task that would have also included the choice of snacks, desserts, breakfast foods, etc. with less variability in environmental impact.

In order to study cross-category substitutions we only included one version of each food product and therefore only one brand to choose from. Products with more familiar brands may have been more chosen by the participants. We observed no difference in familiarity and liking of food products between the two labelling conditions suggesting that the participants selected food products they would have been likely to choose in both conditions, they thus reshaped their food choices within a selection of foods that they liked and were familiar to.

## Conclusions

In the present study, we developed a new traffic-light front-of-pack environmental label based on a score that differentiated food categories. This label was highly noticed, well understood and provided new information that helped the participants to identify the food products of lower environmental impact. Using VR technology that mimicked how an environmental labelling scheme would be likely implemented in real-world supermarkets, we observed a reduction of the environmental impact of food choices in the presence of the environmental label. We thus highlighted that environmental labelling could be a useful tool to shape sustainable dietary patterns at a population level by triggering substitutions across food categories, notably from meat-based to plant-based food products. We also confirmed that VR technology was an effective tool for monitoring individual decision-making behaviours at the point-of-choice in the context of a randomised controlled trial which may be of great interest for future research in behavioural nutrition. Altogether, the results of this study support the idea that environmental labelling could contribute to a global raise in public awareness regarding the environmental impacts of food products across food categories.

## Supplementary Information

**Additional file 1:** **Supplementary Figure S1.** Pictures of the virtual supermarket and of the virtual reality headset and hand controller. **Supplementary Figure S2.** Virtual supermarket shelves. **Supplementary Note S1.** Pop-up message about the environmental label for participants in thelabelling condition. **Supplementary Note S2.** Calculation of the EF single score. **Supplementary Note S3.** Calculation of the five-level environmental score. **Supplementary Note S4.** Design of the environmental label. **Supplementary Table S1.** Deviations from the pre-registered analytic plan. **Supplementary Table S2.** Participants’ characteristics. **Supplementary Table S3.** Fixed effect statistics of linear mixed model testing the effect ofthe label and food choice task on standardised EF single scores in the mainanalysis. **Supplementary Table S4.** Fixed effect statistics of linear mixed models testing the effect ofthe label and food choice task on standardised EF single scores in sensitivityanalyses (with subpopulations, after adjustment for socio-demographic characteristics) or other environmental impact indicators. **Supplementary Table S5.** Fixed effect statistics of linear mixed model testing the effect ofthe label, food choice task and food choice scenario on standardised EF singlescores. **Supplementary Table S6.** Fixed effect statistics of linear mixed models testing the effect ofthe label and food choice task on nutritional quality (FSA scores), energycost, familiarity and liking in the everyday meal scenario. **Supplementary Table S7.** Results from the questionnaire about viewing, understanding and using the environmental label. **Supplementary Table S8.** Results from the questionnaire on the virtual reality experience. 

## Data Availability

The datasets analysed during the current study will be made available on the Open Science Framework project page at the time of publication (https://osf.io/du58k/).

## References

[1] Crippa M, Solazzo E, Guizzardi D, Monforti-Ferrario F, Tubiello FN, Leip A. Food systems are responsible for a third of global anthropogenic GHG emissions. Nat Food [Internet]. Springer US; 2021;2:198–209. Available from: 10.1038/s43016-021-00225-9 10.1038/s43016-021-00225-9 37117443

[2] Clark MA, Domingo NGG, Colgan K, Thakrar SK, Tilman D, Lynch J (2020). Global food system emissions could preclude achieving the 1.5° and 2°C climate change targets. Science.

[3] Willett W, Rockström J, Loken B, Springmann M, Lang T, Vermeulen S (2019). Food in the anthropocene: the EAT–lancet commission on healthy diets from sustainable food systems. Lancet.

[4] Springmann M, Clark M, Mason-D’Croz D, Wiebe K, Bodirsky BL, Lassaletta L, et al. Options for keeping the food system within environmental limits. Nature [Internet]. Springer US; 2018;562:519–25. Available from: 10.1038/s41586-018-0594-0 10.1038/s41586-018-0594-0 30305731

[5] Mertens E, Kuijsten A, van Zanten HH, Kaptijn G, Dofková M, Mistura L, et al. Dietary choices and environmental impact in four European countries. J Clean Prod. 2019;237.

[6] Springmann M, Wiebe K, Mason-D’Croz D, Sulser TB, Rayner M, Scarborough P. Health and nutritional aspects of sustainable diet strategies and their association with environmental impacts: a global modelling analysis with country-level detail. Lancet Planet Heal. 2018;2:e451–61. 10.1016/S2542-5196(18)30206-7 PMC6182055 30318102

[7] Clark MA, Springmann M, Hill J, Tilman D (2019). Multiple health and environmental impacts of foods. Proc Natl Acad Sci U S A.

[8] Hartmann C, Siegrist M (2017). Consumer perception and behaviour regarding sustainable protein consumption: a systematic review. Trends Food Sci Technol.

[9] Macdiarmid JI, Douglas F, Campbell J (2016). Eating like there’s no tomorrow: public awareness of the environmental impact of food and reluctance to eat less meat as part of a sustainable diet. Appetite.

[10] Melendrez-Ruiz J, Chambaron S, Buatois Q, Monnery-Patris S, Arvisenet G (2019). A central place for meat, but what about pulses? studying French consumers’ representations of main dish structure, using an indirect approach. Food Res Int.

[11] ADEME. Affichage environnemental dans le secteur alimentaire : expérimentation 2020/2021. 2022.

[12] Ministère de la transition écologique et solidaire. The anti-waste law in the daily lives of the French people, what does that mean in practice? [Internet]. 2020 [cited 2022 Nov 14]. p. 1–32. Available from: https://www.ecologie.gouv.fr/sites/default/files/en_DPPJL.pdf.

[13] Truelove HB, Parks C (2012). Perceptions of behaviors that cause and mitigate global warming and intentions to perform these behaviors. J Environ Psychol.

[14] Camilleri AR, Larrick RP, Hossain S, Patino-Echeverri D (2019). Consumers underestimate the emissions associated with food but are aided by labels. Nat Clim Chang.

[15] Rondoni A, Grasso S (2021). Consumers behaviour towards carbon footprint labels on food: a review of the literature and discussion of industry implications. J Clean Prod.

[16] Abrahamse W (2020). How to effectively encourage sustainable food choices: a mini-review of available evidence. Front Psychol.

[17] Potter C, Bastounis A, Hartmann-Boyce J, Stewart C, Frie K, Tudor K (2021). The effects of environmental sustainability labels on selection, purchase, and consumption of food and drink products: a systematic review. Environ Behav.

[18] De Bauw M, Matthys C, Poppe V, Franssens S, Vranken L (2021). A combined nutri-score and ‘Eco-Score’ approach for more nutritious and more environmentally friendly food choices? evidence from a consumer experiment in Belgium. Food Qual Prefer.

[19] Vlaeminck P, Jiang T, Vranken L (2014). Food labeling and eco-friendly consumption: experimental evidence from a Belgian supermarket. Ecol Econ.

[20] Jones A, Neal B, Reeve B, Ni Mhurchu C, Thow AM (2019). Front-of-pack nutrition labelling to promote healthier diets: Current practice and opportunities to strengthen regulation worldwide. BMJ Glob Heal.

[21] Bialkova S, van Trijp H (2010). What determines consumer attention to nutrition labels?. Food Qual Prefer.

[22] Hartmann C, Siegrist M. 16 - Virtual reality and immersive approaches to contextual food testing. In: Meiselman HL, editor. Context Eff Environ Prod Des Eval. Woodhead P. 2019. p. 323–38.

[23] van Herpen E, van den Broek E, van Trijp HCM, Yu T (2016). Can a virtual supermarket bring realism into the lab? comparing shopping behavior using virtual and pictorial store representations to behavior in a physical store. Appetite.

[24] Siegrist M, Ung CY, Zank M, Marinello M, Kunz A, Hartmann C (2019). Consumers’ food selection behaviors in three-dimensional (3D) virtual reality. Food Res Int.

[25] Pizzi G, Scarpi D, Pichierri M, Vannucci V (2019). Virtual reality, real reactions?: comparing consumers’ perceptions and shopping orientation across physical and virtual-reality retail stores. Comput Human Behav.

[26] Carrero I, Valor C, Díaz E, Labajo V (2021). Designed to be noticed: a reconceptualization of carbon food labels as warning labels. Sustainability.

[27] Schuldt JP (2013). Does green mean healthy? nutrition label color affects perceptions of healthfulness. Health Commun.

[28] Thøgersen J, Nielsen KS (2016). A better carbon footprint label. J Clean Prod.

[29] Saghaei M (2004). Random allocation software for parallel group randomized trials. BMC Med Res Methodol.

[30] Melendrez-Ruiz J, Goisbault I, Charrier J-C, Pagnat K, Dujourdy L, Arvisenet G (2021). An exploratory study combining eye-tracking and virtual reality: are pulses good “Eye-Catchers” in virtual supermarket shelves?. Front Virtual Real.

[31] ADEME. Agribalyse v3.0 [Internet]. 2020 [cited 2022 Feb 28]. Available from: https://ecolab.ademe.fr/agribalyse

[32] Zampori L, Pant R. Suggestions for updating the Product Environmental Footprint (PEF) method [Internet]. Publ. Off. Eur. Union. Luxembourg; 2019. Available from: https://ec.europa.eu/jrc

[33] Rayner M, Scarborough P, Heart B, Health F. The UK Ofcom Nutrient Profiling Model. UK Ofcom [Internet]. 2009;1–11. Available from: https://www.ndph.ox.ac.uk

[34] Dihr M, Berthold A, Siegrist M, Sütterlin B (2021). Consumers’ knowledge gain through a cross-category environmental label. J Clean Prod.

[35] Fehér A, Gazdecki M, Véha M, Szakály M, Szakály Z (2020). A comprehensive review of the benefits of and the barriers to the switch to a plant-based diet. Sustain.

[36] Cheah I, Sadat Shimul A, Liang J, Phau I (2020). Drivers and barriers toward reducing meat consumption. Appetite.

[37] Michie S, Van Stralen MM, West R (2011). The behaviour change wheel: a new method for characterising and designing behaviour change interventions. Implement Sci.

[38] Hélias A, van der Werf HMG, Soler LG, Aggeri F, Dourmad JY, Julia C (2022). Implementing environmental labelling of food products in France. Int J Life Cycle Assess.

[39] Taufique KMR, Nielsen KS, Dietz T, Shwom R, Stern PC, Vandenbergh MP (2022). Revisiting the promise of carbon labelling. Nat Clim Chang.

[40] Liu T, Wang Q, Su B (2016). A review of carbon labeling: standards, implementation, and impact. Renew Sustain Energy Rev.

[41] Huang Y, Yang X, Li X, Chen Q (2021). Less is better: how nutrition and low-carbon labels jointly backfire on the evaluation of food products. Nutrients.

[42] Aragoncillo L, Orús C (2018). Impulse buying behaviour: An online-offline comparative and the impact of social media. Spanish J Mark - ESIC.

[43] van Herpen E, van den Broek E, van Trijp HCM, Yu T (2016). Can a virtual supermarket bring realism into the lab? comparing shopping behavior using virtual and pictorial store representations to behavior in a physical store. Appetite.

[44] Waterlander WE, Jiang Y, Steenhuis IHM, Ni MC (2015). Using a 3D virtual supermarket to measure food purchase behavior: a validation study. J Med Internet Res.

