# Supplementary material for: Traffic-light front-of-pack environmental labelling across food categories triggers more environmentally friendly food choices: a randomised controlled trial in virtual reality supermarket
Source: Int J Behav Nutr Phys Act. 2023 Jan 26;20:7. doi: 10.1186/s12966-023-01410-8 (PMC9881283; doi:10.1186/s12966-023-01410-8)
Supplement: Supplementary file 1 — Additional file 1: Supplementary Figure S1. Pictures of the virtual supermarket and of the virtual reality headset and hand controller. Supplementary Figure S2. Virtual supermarket shelves. Supplementary Note S1. Pop-up message about the environmental label for participants in thelabelling condition. Supplementary Note S2. Calculation of the EF single score. Supplementary Note S3. Calculation of the five-level environmental score. Supplementary Note S4. Design of the environmental label. Supplementary Table S1. Deviations from the pre-registered analytic plan. Supplementary Table S2. Participants’ characteristics. Supplementary Table S3. Fixed effect statistics of linear mixed model testing the effect ofthe label and food choice task on standardised EF single scores in the mainanalysis. Supplementary Table S4. Fixed effect statistics of linear mixed models testing the effect ofthe label and food choice task on standardised EF single scores in sensitivityanalyses (with subpopulations, after adjustment for socio-demographic characteristics) or other environmental impact indicators. Supplementary Table S5. Fixed effect statistics of linear mixed model testing the effect ofthe label, food choice task and food choice scenario on standardised EF singlescores. Supplementary Table S6. Fixed effect statistics of linear mixed models testing the effect ofthe label and food choice task on nutritional quality (FSA scores), energycost, familiarity and liking in the everyday meal scenario. Supplementary Table S7. Results from the questionnaire about viewing, understanding and using the environmental label. Supplementary Table S8. Results from the questionnaire on the virtual reality experience. [file 12966_2023_1410_MOESM1_ESM.docx]

**Supplementary file**

**Content**

[**Supplementary Figures** 2](#_Toc121500856)

[Supplementary Figure S1: Pictures of the virtual supermarket and of the virtual reality headset and hand controller 2](#_Toc121500857)

[Supplementary Figure S2: Virtual supermarket shelves 4](#_Toc121500858)

[**Supplementary Tables** 5](#_Toc121500859)

[Supplementary Table S1: Deviations from the pre-registered analytic plan 6](#_Toc121500860)

[Supplementary Table S2: Participants’ characteristics 9](#_Toc121500861)

[Supplementary Table S3: Fixed effect statistics of linear mixed model testing the effect of the label and food choice task on standardised EF single scores in the main analysis 10](#_Toc121500862)

[Supplementary Table S4: Fixed effect statistics of linear mixed models testing the effect of the label and food choice task on standardised EF single scores in sensitivity analyses (with subpopulations, after adjustment for socio-demographic characteristics) or other environmental impact indicators 10](#_Toc121500863)

[Supplementary Table S5: Fixed effect statistics of linear mixed model testing the effect of the label, food choice task and food choice scenario on standardised EF single scores 11](#_Toc121500864)

[Supplementary Table S6: Fixed effect statistics of linear mixed models testing the effect of the label and food choice task on nutritional quality (FSA scores), energy cost, familiarity and liking in the everyday meal scenario 11](#_Toc121500865)

[Supplementary Table S7: Results from the questionnaire about viewing, understanding and using the environmental label (N=67)^a^ 12](#_Toc121500866)

[Supplementary Table S8: Results from the questionnaire on the virtual reality experience (N=132) 13](#_Toc121500867)

[**Supplementary Notes** 13](#_Toc121500868)

[Supplementary Note S1: Pop-up message about the environmental label for participants in the labelling condition 13](#_Toc121500869)

[Supplementary Note S2: Calculation of the EF single score 15](#_Toc121500870)

[Supplementary Note S3: Calculation of the five-level environmental score 15](#_Toc121500871)

[Supplementary Note S4: Design of the environmental label 16](#_Toc121500872)

| **Supplementary Figures** |
| --- |

# **Supplementary Figure S1: Pictures of the virtual supermarket and of the virtual reality headset and hand controller**

Screenshots the virtual supermarket shelves

1. Shelf with meal components


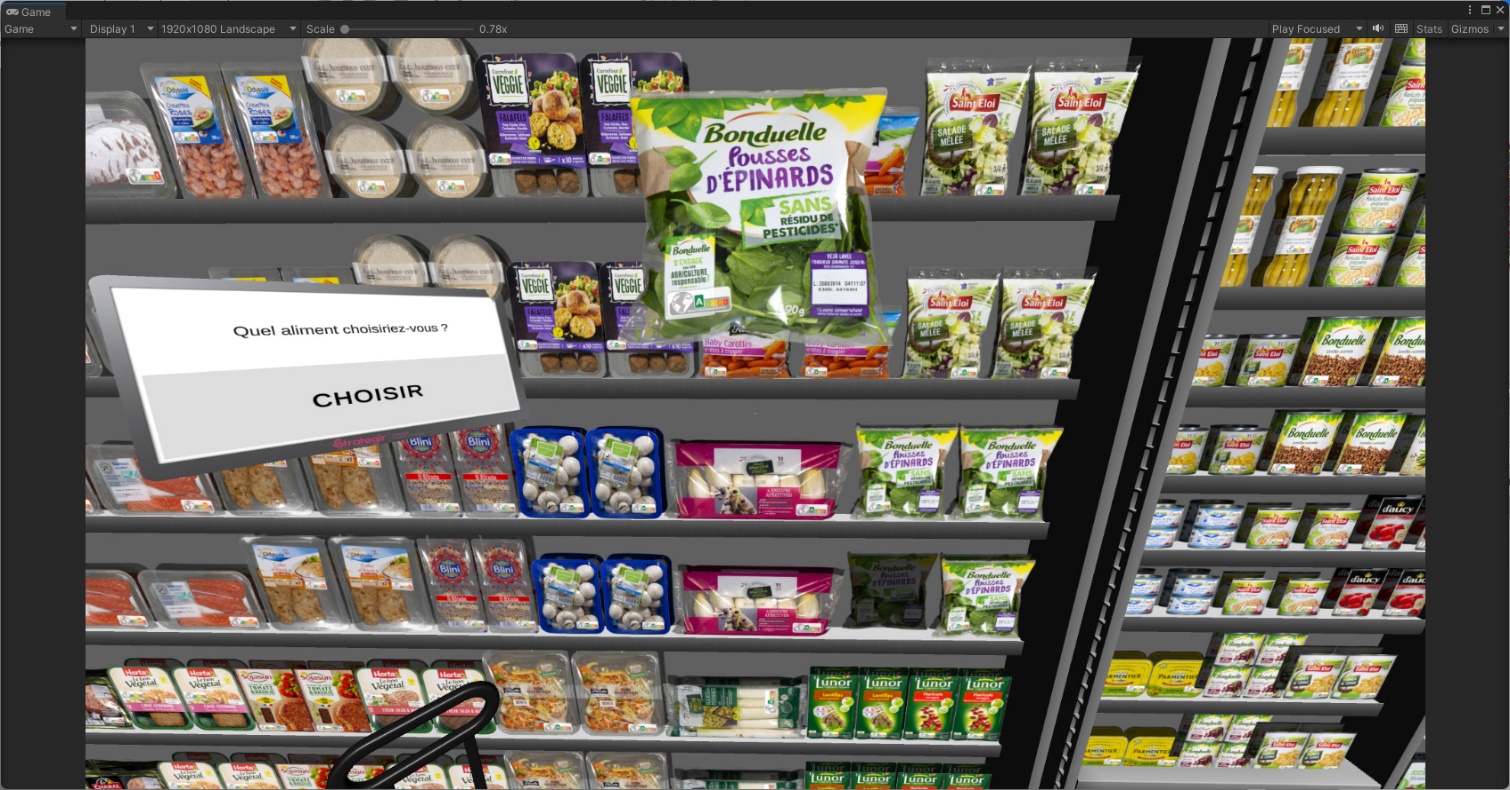


1. Shelf with ready-to-eat meals


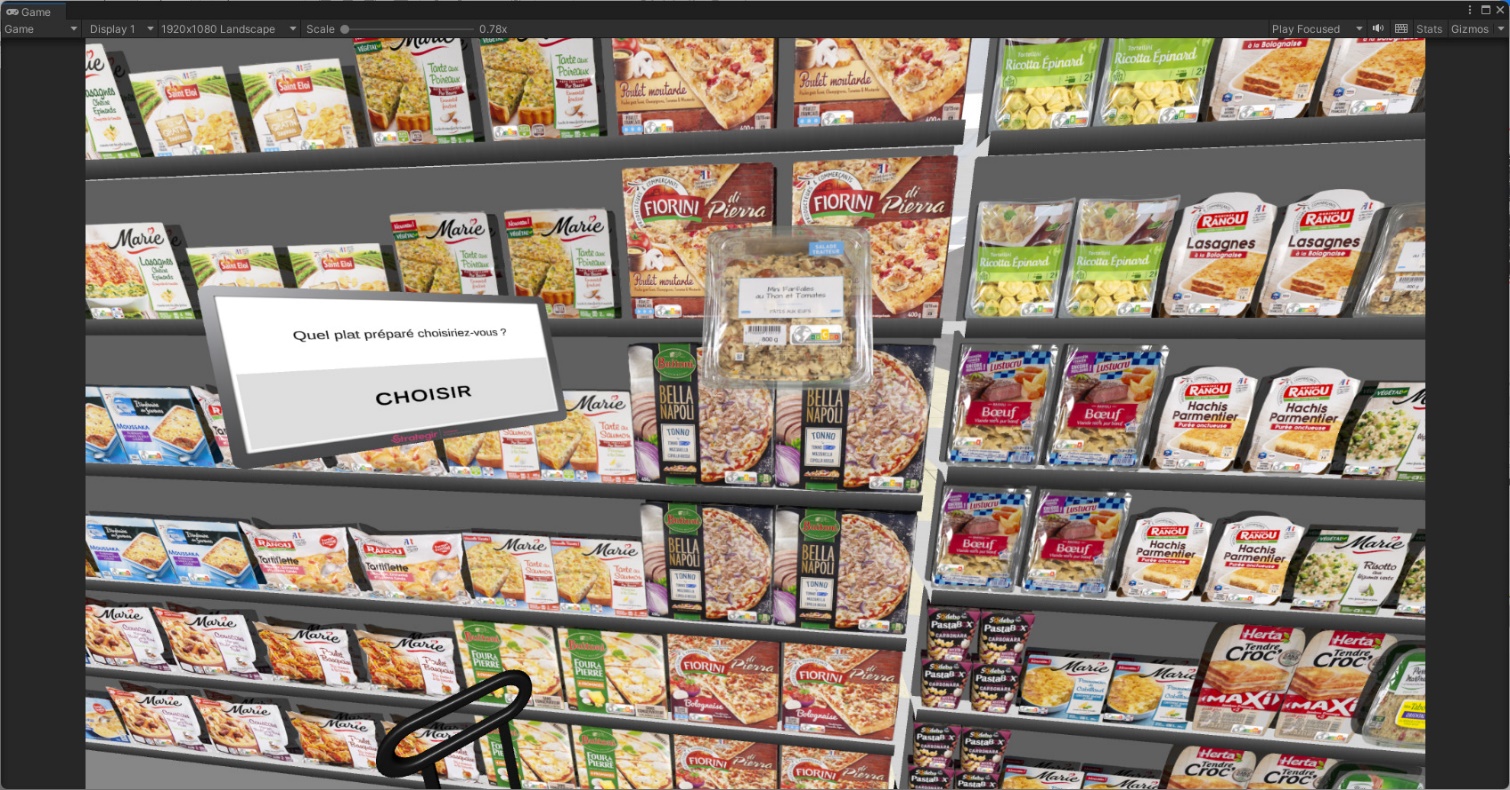


Photographs of the virtual reality headset and hand controller:


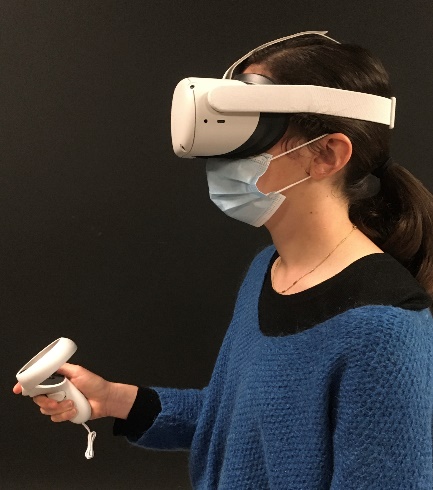

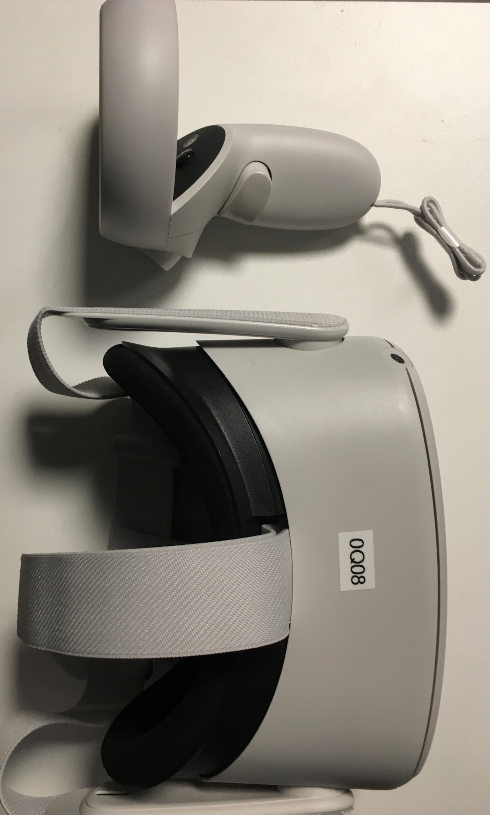


# **Supplementary Figure S2: Virtual supermarket shelves**


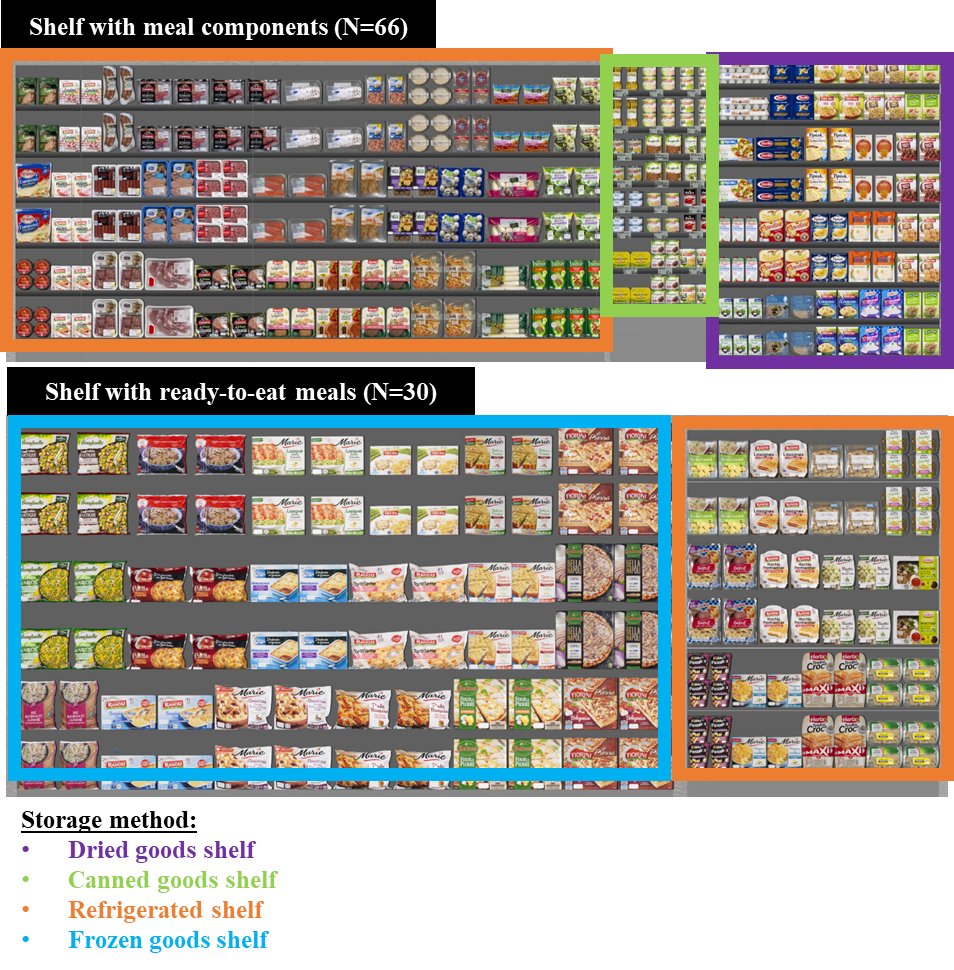


| **Supplementary Tables** |
| --- |

# **Supplementary Table S1: Deviations from the pre-registered analytic plan**

Some methods presented in the paper differ slightly from the descriptions of the pre-registered protocol.

| **Type of analyses** | **Pre-registered analyses** | **Actual analyses** |
| --- | --- | --- |
| Primary outcome | The primary outcome is the environmental impact of the food selected by the participants. The environmental impact of the food selection will be quantified using the EF single score in mPt for 100g of product throughout the life cycle of the products.  The environmental impact of the 3 meal components (i) chosen during task 1 of each scenario is calculated by the mean of the EF single scores of each component (EF_i_)  The environmental impact of the ready-to-eat meal chosen (task 2 of each scenario) is assessed by the EF single score for 100g of this product. | EF single scores, FSA scores and price per kcal of meals were standardised. Standardisation was carried out for the 66 meal components and the 30 ready-to-eat meals separately.  NOVA scores were not included in the paper. |
| Secondary outcomes | - The nutritional quality of the meals chosen by participants is assessed by the FSA score. The nutritional quality of the composed meal (j) chosen during task 1 of each scenario is calculated by the mean of the FSA scores of each of the 3 meal components (i) composing the meal. FSA_i_ scores are calculated for 100g of the meal component i. The nutritional quality of the ready-to-eat meal chosen (task 2 of each scenario) is assessed by the FSA score for 100g of this product. - The liking score of the composed meal (j) is the average of the 3 meal components (i). For the ready-to-eat meal, we will directly use the liking score given by participants. The liking score ranges from 1 to 10, 10 being the highest degree of liking. - The familiarity score of the composed meal is the average of the familiarity score of the 3 meal components selected. The familiarity score of the ready-to-eat meal is directly used. Familiarity scores range from 1 to 5 (1=Never and 5=Very often). - The NOVA score of the composed meal is the average of the NOVA score of the 3 meal components selected. The NOVA score of the ready-to-eat meal will not be analysed since, by definition, all of these food products will be categorised in category 4 (ultra-processed foods). - The price per calorie of the composed meal is the average of the price per calorie of the 3 meal components selected. The price per calorie of the ready-to-eat meal is directly used. |  |
| Primary | - **MODEL 1.1:** Mixed model analysis will be used to test the effect of *labelling* (categorical variable: yes or no), *food choice task* (categorical variable: composed or ready-to-eat meal) and *labelling* food choice task interaction* on EF single scores in the “everyday meal scenario”, with random effect of participants to account for correlation between repeated measures. If the *labelling* food choice task interaction* is not significant, it will be removed from the model. - **MODEL 1.2:** Mixed model analysis will be used to test the effect of *labelling* (categorical variable: yes or no), *food choice task* (categorical variable: composed or ready-to-eat meal), *food choice scenario* (categorical variable: “everyday meal scenario” or “environmentally friendly meal scenario”), *labelling*scenario*, *labelling* food choice task* and *scenario* food choice task* interactions on EF single scores, with random effect of participants to account for correlation between repeated measures. Non-significant interactions will be removed from the model. | No deviation |
| Sensitivity analysis | - Sensitivity analysis will be carried out to analyse if the results found for **MODEL 1.1.** remain the same when aim guessers are excluded. - **MODEL 1.1.** will be adjusted for age, sex, level of education and BMI. - **MODEL 1.1.** (raw and adjusted) will be replicated for the GHGe from the Agribalyse database instead of the EF single score. | Sensitivity analysis also excluded outliers.  Raw and adjusted MODEL 1.1 were also replicated for ozone depletion and particulate matter indicators. |
| Secondary (secondary outcomes) | **MODEL 2.1:** Mixed model analysis will be used to test the effect of *labelling* (categorical variable: yes or no), *food choice task* (categorical variable: composed or ready-to-eat meal) and *labelling* food choice task* interaction on 4 dependent variables (FSA scores, Price per kcal, liking and familiarity scores), with random effect of participants to account for correlation between repeated measures. We will only look at the data from the first scenario (“everyday meal scenario”).  ANOVA model will be used to test the effect of *labelling* (categorical variable: yes or no) on NOVA score for task 1 (for the 3 meal components chosen). We will only look at the data from the first scenario (“everyday meal scenario”). | Results for level of transformation are not presented in this paper. |
| Secondary (ethic motives) | **MODEL 2.2:** Mixed model analysis will be used to test the effect of *labelling* (categorical variable: yes or no), *food choice task* (categorical variable: composed or ready-to-eat meal), *labelling* food choice task* interaction, *Ethic_score* (continuous variable) and *labelling* Ethic_score* interaction on EF single scores, with random effect of participants to account for correlation between repeated measures. If the *labelling* food choice task* interaction is not significant, it will be removed from the model. | Results from this model are not presented in the paper. |
| Exploratory | See pre-registered protocol for extensive list of exploratory analyses. | All exploratory analyses listed in the pre-registered protocol were carried out. Only exploratory analyses relative to manipulation checks are presented in this paper.  We added results from VR-tracking data since we thought it would be interesting to check if participants in the two labelling conditions had the same visual exploratory behaviour. |

# **Supplementary Table S2: Participants’ characteristics**

|  | **Overall** (N=132) | **No Label** (N=65) | **Environmental Label** (N=67) | **p-value**^a^ |
| --- | --- | --- | --- | --- |
| **Age**, years*, mean ± SD* | 43.6 ± 12.1 | 43.5 ± 12.0 | 43.7 ± 12.4 | 0.933 |
| **Gender**, female, *n (%)* | 67 (50.8%) | 34 (52.3%) | 33 (49.3%) | 0.726 |
| **Employment status*,*** *n (%)* |  |  |  | 0.371 |
| Full time or part time | 103 (78%) | 52 (80%) | 51 (76.1%) |  |
| Student | 10 (7.6%) | 5 (7.7%) | 5 (7.5%) |  |
| Retired | 10 (7.6%) | 6 (9.2%) | 4 (6%) |  |
| Looking after home | 2 (1.5%) | 1 (1.5%) | 1 (1.5%) |  |
| Looking for a job | 5 (3.8%) | 0 (0%) | 5 (7.5%) |  |
| Other | 2 (1.5%) | 1 (1.5%) | 1 (1.5%) |  |
| **Highest educational qualification*,*** *n (%)* |  |  |  | 0.759 |
| < High school + 2-year diploma | 32 (24.2%) | 17 (26.2%) | 15 (22.4%) |  |
| High school + 2-year diploma | 32 (24.2%) | 16 (24.6%) | 16 (23.9%) |  |
| High school + 3/4-year diploma | 30 (22.7%) | 16 (24.6%) | 14 (20.9%) |  |
| ≥ High school + 5-year diploma | 38 (28.8%) | 16 (24.6%) | 22 (32.8%) |  |
| **Financial status,** *n (%)* |  |  |  | 0.759 |
| You are at ease | 29 (22%) | 13 (20%) | 16 (23.9%) |  |
| It’s okay | 70 (53%) | 34 (52.3%) | 36 (53.7%) |  |
| It’s tight, you need to be careful | 30 (22.7%) | 17 (26.2%) | 13 (19.4%) |  |
| You make it to the end of the month with difficulties | 2 (1.5%) | 1 (1.5%) | 1 (1.5%) |  |
| You cannot make it to the end of the month without debts | 1 (0.8%) | 0 | 1 (1.5%) |  |
| **Number of adults in the household***, mean ± SD* | 2.14 ± 1,03 | 2.08 ± 1,03 | 2.21 ± 1,02 | 0.462 |
| **Number of children in the household (<14 years old)***, mean ± SD* | 0.57 ± 0,83 | 0.58 ± 0,86 | 0.57 ± 0,8 | 0.904 |
| **BMI***, mean ± SD* | 24.4 ± 4.0 | 24.4 ± 3.5 | 24.4 ± 4.5 | 0.995 |
| **Dieting status**, yes, *n (%)* | 13 (9.8%) | 5 (7.6%) | 8 (11.9%) | 0.413 |

^a^ Student t-tests or Chi-Square test of independence were used to test statistical significance between labelling conditions for each socio-demographic variable.

# **Supplementary Table S3: Fixed effect statistics of linear mixed model testing the effect of the label and food choice task on standardised EF single scores in the main analysis**

| **Model** | **Type III tests** | | **Estimate^*^** | **95% LCL^*^** | **95%UCL^*^** |
| --- | --- | --- | --- | --- | --- |
|  | **F** | **p** |  |  |  |
| **Complete (n=132)** | | | | | |
| *(Intercept)* |  |  | 0.04 | -0.09 | 0.16 |
| *Labelling* | 4.93 | 0.028 | -0.17 | -0.32 | -0.02 |
| *Food choice task* | 28.64 | <.0001 | -0.37 | -0.50 | -0.23 |
| *Labelling* Food choice task* | 2.59 | 0.110 |  |  |  |

^a^ Intercept estimate for the reference group: No label and task 1. Estimates of the fixed effects must be interpreted as differences from the reference group.

*Estimates and confidence intervals were calculated after removing non-significant interaction from the mixed model.

# **Supplementary Table S4: Fixed effect statistics of linear mixed models testing the effect of the label and food choice task on standardised EF single scores in sensitivity analyses (with subpopulations, after adjustment for socio-demographic characteristics) or other environmental impact indicators**

| **Model** | **Type III tests** | | **Estimate^*^** | **95% LCL^*^** | **95%UCL^*^** |
| --- | --- | --- | --- | --- | --- |
|  | **F** | **p** |  |  |  |
| **Model 1 (EF single score) without aim-guessers (n=121)** | | | | | |
| *(Intercept)* |  |  | 0.02 | -0.11 | 0.15 |
| *Labelling* | 4.63 | 0.033 | -0.17 | -0.34 | -0.01 |
| *Food choice task* | 23.31 | <.0001 | -0.34 | -0.48 | -0.20 |
| *Labelling* Food choice task* | 1.72 | 0.192 |  |  |  |
| **Model 2 (EF single score) without outliers (n=127)** | | | | | |
| *(Intercept)* |  |  | 0.05 | -0.08 | -0.18 |
| *Labelling* | 5.36 | 0.022 | -0.18 | -0.34 | -0.03 |
| *Food choice task* | 26.11 | <.0001 | -0.37 | -0.51 | -0.22 |
| *Labelling* Food choice task* | 2.54 | 0.113 |  |  |  |
| **Model 3 (EF single score) adjusted for socio-demographic characteristics (gender, age, BMI and highest educational qualification) (n=132)** | | | | | |
| *(Intercept)* |  |  | -0.10 | -0.64 | 0.45 |
| *Labelling* | 5.68 | 0.019 | -0.18 | -0.33 | -0.03 |
| *Food choice task* | 28.64 | <.0001 | -0.36 | -0.50 | -0.23 |
| *Labelling* Food choice task* | 2.59 | 0.110 |  |  |  |
| **Model 4 (dependant variable = GHGE) (n=132)** | | | | | |
| *(Intercept)* |  |  | 0.03 | -0.12 | 0.19 |
| *Labelling* | 3.96 | 0.049 | -0.18 | -0.36 | -0.00 |
| *Food choice task* | 4.51 | 0.036 | -0.18 | -0.35 | -0.01 |
| *Labelling* Food choice task* | 2.74 | 0.101 |  |  |  |
| **Model 5 (dependant variable = ozone depletion) (n=132)** | | | | | |
| *(Intercept)* |  |  | -0.11 | -0.20 | -0.03 |
| *Labelling* | 0.11 | 0.744 | -0.02 | -0.12 | 0.09 |
| *Food choice task* | 22.69 | <.0001 | -0.21 | -0.30 | -0.12 |
| *Labelling* Food choice task* | 1.51 | 0.221 |  |  |  |
| **Model 6 (dependant variable = particulate matter) (n=132)** | | | | | |
| *(Intercept)* |  |  | 0.03 | -0.12 | 0.17 |
| *Labelling* | 4.04 | 0.047 | -0.17 | -0.34 | 0.00 |
| *Food choice task* | 8.74 | 0.004 | -0.23 | -0.39 | -0.08 |
| *Labelling* Food choice task* | 2.11 | 0.148 |  |  |  |

^a^ Intercept estimate for the reference group: No label and task 1. Estimates of the fixed effects must be interpreted as differences with the reference group.

*Estimates and confidence intervals were calculated after removing non-significant interaction from the mixed model.

# **Supplementary Table S5: Fixed effect statistics of linear mixed model testing the effect of the label, food choice task and food choice scenario on standardised EF single scores**

| **Model** | **Type III tests** | | **Estimate^*^** | **95% LCL^*^** | **95%UCL^*^** |
| --- | --- | --- | --- | --- | --- |
|  | **F** | **p** |  |  |  |
| **Complete (n=132)** | | | | | |
| *(Intercept)* |  |  | 0.029 | -0.07 | 0.12 |
| *Labelling* | 11.80 | 0.001 | -0.18 | -0.28 | -0.08 |
| *Food choice task* | 65.25 | <0.001 | -0.34 | -0.42 | -0.26 |
| *Food choice scenario* | 54.68 | <0.001 | -0.31 | -0.39 | -0.23 |
| *Labelling*Food choice task* | 2.11 | 0.148 |  |  |  |
| *Labelling*Scenario* | 0.06 | 0.805 |  |  |  |
| *Scenario*Food choice task* | 0.36 | 0.551 |  |  |  |

^a^ Intercept estimate for the reference group: No label, task 1 and everyday scenario. Estimates of the fixed effects must be interpreted as differences with the reference group.

*Estimates and confidence intervals were calculated after removing non-significant interactions from the mixed model.

# **Supplementary Table S6: Fixed effect statistics of linear mixed models testing the effect of the label and food choice task on nutritional quality (FSA scores), energy cost, familiarity and liking in the everyday meal scenario**

| **Models** | **Type III tests** | | **Estimate^*^** | **95% LCL^*^** | **95%UCL^*^** |
| --- | --- | --- | --- | --- | --- |
| **(n=132)** | **F** | **p** |  |  |  |
| **FSA scores** | | | | | |
| *(Intercept)* |  |  | -0.06 | -0.22 | 0.10 |
| *Labelling* | 1.66 | 0.200 | -0.12 | -0.30 | 0.06 |
| *Food choice task* | 13.97 | <0.001 | -0.33 | -0.51 | -0.16 |
| *Labelling* Food choice task* | 0.47 | 0.494 |  |  |  |
| **Energy cost** | | | | | |
| *(Intercept)* |  |  | 0.28 | 0.08 | 0.48 |
| *Labelling* | 0.73 | 0.395 | 0.10 | -0.13 | 0.33 |
| *Food choice task* | 9.14 | 0.003 | -0.36 | -0.59 | -0.12 |
| *Labelling* Food choice task* | 0.03 | 0.862 |  |  |  |
| **Familiarity scores** | | | | | |
| *(Intercept)* |  |  | 3.98 | 3.79 | 4.18 |
| *Labelling* | 0.15 | 0.696 | -0.05 | -0.28 | 0.19 |
| *Food choice task* | 96.59 | <0.001 | -1.07 | -1.28 | -0.85 |
| *Labelling* Food choice task* | 0.53 | 0.467 |  |  |  |
| **Liking scores** | | | | | |
| *(Intercept)* |  |  | 8.33 | 8.03 | 8.63 |
| *Labelling* | 0.03 | 0.868 | 0.03 | -0.35 | 0.41 |
| *Food choice task* | 48.73 | <0.001 | -0.96 | -1.24 | -0.69 |
| *Labelling* Food choice task* | 2.4 | 0.124 |  |  |  |

^a^ Intercept estimate for the reference group: No label and task 1. Estimates of the fixed effects must be interpreted as differences with the reference group.

*Estimates and confidence intervals were calculated after removing non-significant interaction from the mixed model.

# **Supplementary Table S7: Results from the questionnaire about viewing, understanding and using the environmental label (N=67)^a^**

| **Observation: *How many of the products you chose had an environmental label?*** | | | | |
| --- | --- | --- | --- | --- |
|  | I don’t know | Less than half of the chosen products | More than half of the chosen products | All of the chosen products |
| % | 11 | 6 | 16 | 67 |
| **Understanding: According to you, this label [image of the environmental label] describes…** | | | | |
|  | The nutritional quality of food products | The environmental impact of food products | The nutritional quality and environmental impact of food products | |
| % | 2 | 95 | 3 | |
| **Use: When asked to compose a meal, how useful was the environmental label?**  **[score between 0 and 100]** | | | | |
|  | Mean | SD |  |  |
| Everyday meal scenario | 41 | *29* | **Paired t-test:**  *p-value* = 0.053 | |
| Environmentally friendly meal scenario | 82 | *26* |  |  |

^a^ Participants in the labelling condition

# **Supplementary Table S8: Results from the questionnaire on the virtual reality experience (N=132)**

| **Questionnaire items** | **Disagree^a^**  **(%)** | **Agree^b^**  **(%)** |
| --- | --- | --- |
| I found it easy to move around in the virtual supermarket. | 2 | 98 |
| I found it easy to select food products and to place them in the shopping basket. | 2 | 98 |
| I was not disturbed by what was happening around me / outside the virtual supermarket. | 0 | 100 |
| I felt involved in the choice of food products I made. | 2 | 98 |
| The food products found in the virtual supermarket were food products commonly found in real supermarkets. | 4 | 96 |
| The food products I chose in the virtual supermarket were food products that I could choose in real life. | 2 | 98 |

^a^ Percentage of participants who answered “strongly disagree” or “disagree” to the statement

^b^ Percentage of participants who answered “strongly agree” or “agree” to the statement

| **Supplementary Notes** |
| --- |

# **Supplementary Note S1: Pop-up message about the environmental label for participants in the labelling condition**

Sixty-seven participants were allocated to the environmental label condition. Before performing the four food choice tasks, these participants saw once a pop-up message explaining the front-of-pack environmental label. This explanation was accompanied with images of the environmental label. The pop-up message is below (translated from French to English).

| An environmental label is present on each food product of the virtual supermarket.  It describes if its production had a negative impact on the environment (emissions of greenhouse gases, water consumption, land use, etc.)  Food products are rated with an environmental score ranging from **A** to **E**:  **A** indicates the lowest environmental impact and **E** the highest environmental impact.  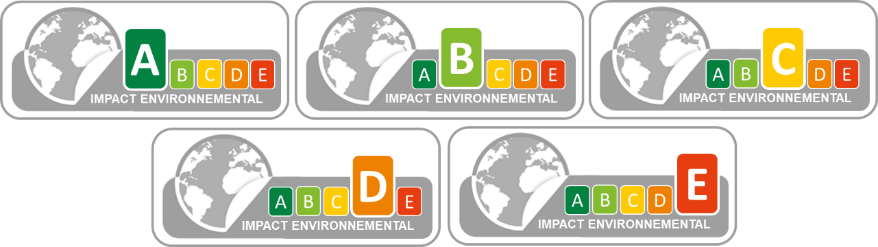 |
| --- |

# **Supplementary Note S2: Calculation of the EF single score**

The European Commission recommends the use of the EF single score when studying the environmental impact of food products taking into account the 16 indicators, calculated throughout the life cycle of the products : climate change (kg CO_2_ eq), ozone depletion (kg CFC-11 eq), human toxicity (cancer and non-cancer effects) (CTUh), particulate matter (disease incidence), ionizing radiation (kBq U235 eq), photochemical ozone formation (kg NMVOC eq), marine eutrophication (kg N eq), freshwater eutrophication (kg P eq), terrestrial eutrophication (mol N eq), freshwater ecotoxicity (CTUe), land use (point), water use (m3 world eq), minerals and metals use (kg Sb eq), fossil use (MJ) and acidification (mol H^+^ eq) [1]. In order to obtain a single EF score, the indicators are weighed according to a method described by the European Commission^1^. EF single scores for the foods present in the Agribalyse database are expressed in mPt^[[1]](#footnote-1)^ for 1 kg of product.

# **Supplementary Note S3: Calculation of the five-level environmental score**

To create the environmental label for each of the 96 food products of our virtual supermarket, we assigned to each product an environmental score segmented into five levels. We defined the cut-offs across levels as the quintile values from a larger selection of similar foods from the Agribalyse database. For the first food choice task (choice of 3 meal components to compose a meal), we created quintiles for the EF score of 1655 foods that correspond to the food categories included in the virtual supermarket (“Fruits, vegetables, legumes, nuts and seeds”, “Cereal products”, “Milk and dairy products” and “Meat, egg and fish”). We replicated this method for the second food choice task (choice of a ready-to-eat meal among 30 products) by calculating quintiles among 281 foods. The cut-off values of the quintiles calculated for the two tasks separately are displayed in the table below.

| **Cut-off values for meal components**  *(EF scores in mPt/kg of product)* | **Cut-off values for ready-to-eat meals**  *(EF scores in mPt/kg of product)* |
| --- | --- |
| across 1655 products | across 281 products |
| 0.05 < **Q1** ≤ 0.17 | 0.13 < **Q1** ≤ 0.20 |
| 0.17 < **Q2** ≤ 0.35 | 0.20 < **Q2** ≤ 0.36 |
| 0.35 < **Q3** ≤ 0.62 | 0.36 < **Q3** ≤ 0.46 |
| 0.62 < **Q4** ≤ 1.36 | 0.46 < **Q4** ≤ 0.84 |
| 1.36 < **Q5** ≤ 6.09 | 0.84 < **Q5** ≤ 3.23 |

The process for the creation of the environmental score for the first food choice task (meal component shelf) is summarised in the figure below.


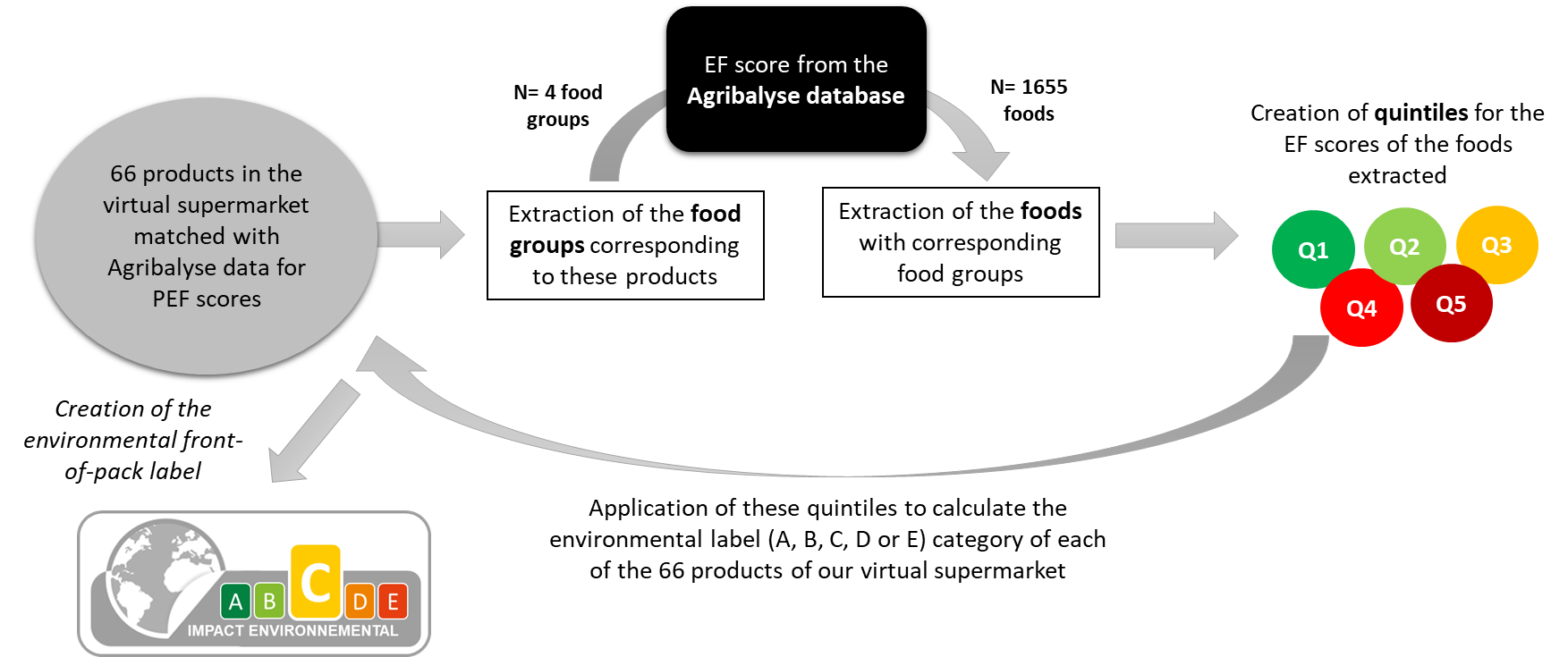


**Supplementary Note S4: Design of the environmental label**

We described above how the environmental score of the food products was calculated. It was also necessary to define a way to display this score on the food products in the virtual supermarket. No environmental front-of-pack label had reached a consensus in France at the time of the study (May 2021). Many countries, such as UK or Australia, have already adopted carbon labels for various products but their effectiveness has been questioned in previous studies [2,3]. One reason advocated to explain this inefficiency was the difficulty to understand these labels. In order to overcome the cognitive and motivational barriers that lessen the effectiveness of labels, a review highlighted the three main points to take into account when designing an environmental label [4]:

1. The label needs to provide a reference. If raw information is provided to consumers, they find it difficult to understand its meaning. Consumers are unable to evaluate what is a high or a low carbon footprint if they are indicated as absolute values [5].
2. The label needs to be prescriptive. It needs to provide procedural knowledge. It is important that participants can, in a glimpse, compare the environmental impact of two products. This characteristic was also described in a study that asked participants to rank six different environmental labels from the most to the least understandable [6].
3. The label needs to be simple. There is a trade-off to find between giving enough information for consumers to know what the label is referring to, while avoiding unnecessary information that could lead to a cognitive overload.

The ideal label proposed by Carrero et al., 2021 to answer simultaneously these three aspects is a “traffic-light label”. Indeed, it has been shown that green is often seen as a validation (“go”) and a positive colour whereas red is associated with negative aspects and danger [7]. Moreover, a study testing the same carbon label in the form of a traffic light (green, yellow or red footprint) or with only a black footprint showed an increase effectiveness when using the traffic-light colours [8].

For the present study, we decided to adapt an existing label to meet the requirements mentioned above. In September 2020, the French agency for ecological transition (ADEME) published a design for a label depicting the environmental impact from life cycle analysis of products or services [9], see figure below. The colour was changed to obtain a “traffic light label” since it is considered as the best option to increase understanding and behaviour change in consumers. We determined the placement and the size of the environmental label on the products. These characteristics had been studied in focus groups revealing the importance of having the label on the front of the product and in a sufficient size, allowing it to be easily seen by participants when they are standing in the aisles. A study analysing the “horizontal location effect” of carbon labels revealed that placing the label on the right of the product increases the intended purchases of foods with a good environmental impact [10]. Thus, we decided to place the environmental label on the bottom right hand corner of the food products as shown in the figure below. The size of our environmental label was proportionate to the size of the food product and was the same size as the Nutri-Score label already displayed on some food products in France.


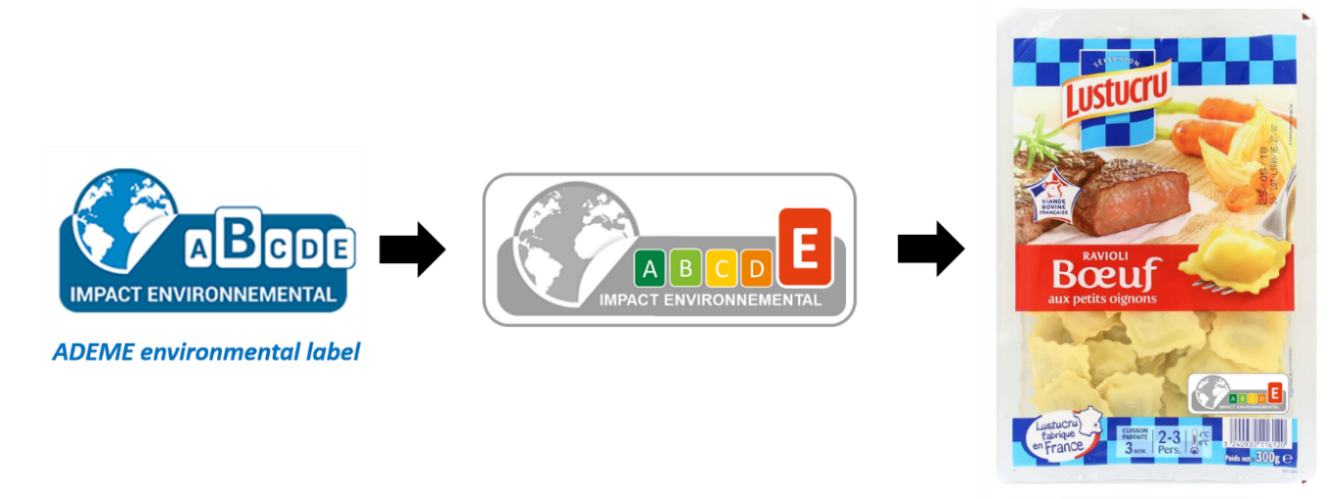


**References**

1. Zampori L, Pant R. Suggestions for updating the Product Environmental Footprint (PEF) method [Internet]. Publ. Off. Eur. Union. Luxembourg; 2019. Available from: https://ec.europa.eu/jrc

2. Gadema Z, Oglethorpe D. The use and usefulness of carbon labelling food: A policy perspective from a survey of UK supermarket shoppers. Food Policy [Internet]. Elsevier Ltd; 2011;36:815–22. Available from: http://dx.doi.org/10.1016/j.foodpol.2011.08.001

3. Grunert KG, Hieke S, Wills J. Sustainability labels on food products: Consumer motivation, understanding and use. Food Policy [Internet]. Elsevier Ltd; 2014;44:177–89. Available from: http://dx.doi.org/10.1016/j.foodpol.2013.12.001

4. Carrero I, Valor C, Díaz E, Labajo V. Designed to be noticed: A reconceptualization of carbon food labels as warning labels. Sustainability. 2021;13:1–14.

5. Larrick RP, Soll JB, Keeney RL. Designing better energy metrics for consumers. Behav Sci Policy. 2015;1:63–75.

6. Vlaeminck P, Jiang T, Vranken L. Food labeling and eco-friendly consumption: Experimental evidence from a Belgian supermarket. Ecol Econ [Internet]. Elsevier B.V.; 2014;108:180–90. Available from: http://dx.doi.org/10.1016/j.ecolecon.2014.10.019

7. Schuldt JP. Does Green Mean Healthy? Nutrition Label Color Affects Perceptions of Healthfulness. Health Commun. 2013;28:814–21.

8. Thøgersen J, Nielsen KS. A better carbon footprint label. J Clean Prod [Internet]. Elsevier Ltd; 2016;125:86–94. Available from: http://dx.doi.org/10.1016/j.jclepro.2016.03.098

9. ADEME. Affichage environnemental dans le secteur alimentaire : expérimentation 2020/2021 [Internet]. 2020 [cited 2021 Feb 9]. Available from: https://www.ademe.fr/expertises/consommer-autrement/passer-a-laction/reconnaitre-produit-plus-respectueux-lenvironnement/dossier/laffichage-environnemental/affichage-environnemental-secteur-alimentaire-experimentation-20202021

10. Zhou S, Wang H, Li S, Chen Y, Wu J. Carbon labels and “horizontal location effect”: Can carbon labels increase the choice of green product? Glob Ecol Conserv [Internet]. Elsevier Ltd; 2019;18:e00609. Available from: https://doi.org/10.1016/j.gecco.2019.e00609

1. Pt means “eco-indicator point” and on a scale, 1 mPt represents the mean annual environmental load of an average European inhabitant. [↑](#footnote-ref-1)
